# Wedelactone-loaded exosomes for sepsis-induced liver injury: a novel therapeutic strategy

**DOI:** 10.1080/10717544.2026.2693353

**Published:** 2026-06-30

**Authors:** Yanping Yin, Lulu Zhang, Yanli Yin, Jinyi Zhao, Rui Gong, Xuan Zhou, Haiyue Zhang, Fei Mu, Jingwen Wang

**Affiliations:** a Department of Pharmacy, Xijing Hospital, The Fourth Military Medical University, Xi'an, China; b Zhejiang Garden Nutrition Technology Co., Ltd., Jin'hua, China; c Department of Health Statistics, Ministry of Education Key Lab of Hazard Assessment and Control in Special Operational Environment, School of Public Health, Air Force Medical University, Xi'an, Shaanxi, China

**Keywords:** Sepsis-induced liver injury, wedelactone, exosome, oxidative stress, ferroptosis

## Abstract

Sepsis-induced liver injury (SILI) is an important cause of death in intensive care patients, which seriously affects clinical prognosis. Wedelolactone (WEL) exhibits hepatoprotective properties, however, its clinical application is constrained by its poor solubility and insufficient targeting ability. Therefore, in this study, an innovative exosome (Exo)-based drug delivery system loaded with WEL (Exo@WEL) was constructed. The aim was to enhance the liver-targeting efficacy and therapeutic performance of WEL. Exo were extracted from the mice macrophages cell line RAW264.7 by differential centrifugation, and WEL was successfully loaded using ultrasonic incubation. Exo@WEL was characterized by TEM, particle size analysis, and NTA, confirming its structural suitability as an exogenous agent. DiR labeling revealed that Exo@WEL had a significantly enhanced liver-targeting ability compared to WEL. Safety was confirmed by HE staining test. In the SILI models, Exo@WEL showed better hepatoprotection over WEL. Beyond entinfinmtory actvity menifested y decreased ro infammatory rokines, Exo@WEL reinforced antioxidant function and efectively restained feroptosis. Importantly, pharmacological inhibition of ML385, a selective Nrf2 inhibitor, confirmed the critical regulatory role of the Nrf2 pathway in mediating these multifaceted liver protective effects. This study pioneered the development of a targeted Exo-based nanoplatform (Exo@WEL) for SILI therapy. The mechanism study has revealed a potential therapeutic strategy for inhibiting oxidative stress and ferroptosis by regulating the Nrf2/SLC7A11/GPX4 axis. This preparation provides a novel nanotherapeutic strategy that combines high efficiency and safety for the treatment of SILI. These findings also lay a theoretical foundation for the clinical translation of Exo drug delivery systems.

## 1. Introduction

Sepsis is a life-threatening systemic syndrome characterized by a dysregulated host response to infection. This condition results in profound immune dysfunction and multi-organ failure. As a major global health burden, it remains the primary cause of mortality in intensive care settings (Strunk et al. 2024; Aissaoui et al. 2025). Persistent inflammation, immunosuppression, and catabolic syndrome in the later stage of sepsis are the main causes of death (Angus et al. 2001; Venet and Monneret 2018). About a quarter of sepsis patients cannot survive hospitalization (Nesseler et al. 2012; Marshall 2014). In 2019, sepsis remained the leading cause of death worldwide, but there were significant regional differences in mortality rates. Specifically, in high-income countries, the in-hospital mortality rate based on the Sepsis-3 definition is approximately 15%–25% (Fleischmann-Struzek et al. 2020; Li et al. 2023). However, in middle- and low-income countries, owing to limitations in access to medical resources, the median in-hospital mortality rate for adult sepsis can be as high as 30%, and in some extremely resource-poor environments, it can even exceed 50% (Markwart et al. 2020). Thus, it poses a serious threat to society and medical care. The liver has abundant blood circulation. During systemic infection, the liver plays a key role in sepsis by regulating immune and metabolic processes (Nesseler et al. 2012; Strnad et al. 2017). Liver injury has an important impact on the severity and clinical outcome of sepsis patients (Abdelnaser et al. 2023). Apart from the liver, sepsis often leads to multiple organ dysfunction, among which acute kidney injury, cardiac injury, and lung injury are common and serious complications that affect prognosis (Üstündağ et al. 2023; Abdelnaser et al. 2024). Studies have shown that drugs can alleviate sepsis-induced kidney injury by regulating pathways such as Nrf2/HO-1, apoptosis, and oxidative stress (Abdelnaser et al. 2024; 2025). Meanwhile, protective strategies targeting the myocardium and lung tissues, such as melatonin combined with ascorbic acid, coenzyme Q10, and vitamin C, etc., have also been shown effects in improving heart and lung injuries in experimental models (Üstündağ et al. 2023; 2023). Therefore, intervention targeting the treatment targets of liver injury caused by sepsis while also considering protection of multiple organs, such as the heart, kidneys, and lungs, are expected to provide a more comprehensive treatment strategy for improving the overall prognosis of sepsis patients.

Exo are a subset of extracellular vesicles derived from endosomes (Kim et al. 2024). It is rich in a variety of proteins, amino acids, nucleic acids, lipids, and metabolites, which affect the biological function of recipient cells by transferring these bioactive substances to recipient cells (Kalluri and LeBleu 2020). The effects of Exo on the immune system include antigen presentation, maturation, differentiation and activation of immune cells, etc. Their role as carriers of immunotherapy drugs has been extensively studied (Xie et al. 2024). There is a large amount of evidence indicating that Exo are expected to become important targets and methods for treating liver injury in sepsis. For example, mesenchymal stem cell-derived Exo alleviates sepsis-induced acute liver injury by suppressing MALAT1 through microRNA-26a-5p: an innovative immunopharmacological intervention and therapeutic approach for sepsis (Cai et al. 2023). BMSCs-derived Exo attenuates septic liver injury by inhibiting macrophage STING signaling (Liu et al. 2023). Stem cell-derived Exo can ameliorate the destructive effects of inflammation caused by sepsis by reducing the levels of inflammatory factors and tissue damage (Eshghi et al. 2022). Although current studies provide evidence for Exo treatment of sepsis, the mechanism remains to be fully explored.

In recent years, an increasing number of studies have shown that oxidative stress has a major impact on sepsis-induced liver injury (SILI) (Xiao et al. 2022; Mohyeldin et al. 2023). Oxidative stress represents an imbalance between pro-oxidant and antioxidant mechanisms within the body. It triggers a cascade of pathological reactions involving lipid peroxidation, depletion of antioxidant reserves, and disruption of functional signaling pathways. (Li et al. 2021; Teixeira et al. 2024; Zhang et al. 2024). When liver cells are stimulated, ferroptosis can be inhibited, and inflammatory infiltration of liver tissue can be reduced (Liu et al. 2023). Ferroptosis is a type of ferroptosis-mediated necrotizing cell death. Oxidative stress initiates and mediates it, and ferroptosis inhibition can protect SILI (Huang et al. 2024; Xu et al. 2025). Ferroptosis is characterized by iron-dependent lipid radical accumulation and inactivation of the key antioxidant enzyme glutathione peroxidase 4 (GPX4) (Song et al. 2024; Yi et al. 2025). Nuclear factor-E2-related factor 2 (Nrf2) is a critical transcription factor required for maintaining cellular oxidative stability and redox homeostasis. It serves as a key link between oxidative stress and ferroptosis by modulating the expression of related proteins. Nrf2 influences both oxidative stress responses and ferroptotic pathways. Nrf2 is a pivotal transcription factor essential for maintaining oxidative balance and cellular redox homeostasis. Activation of the Nrf2 signaling pathway exerts hepatoprotective effects against SILI. Mechanistically, this protection involves upregulation of the SLC7A11/GPX4 axis, which mitigates oxidative damage (Deng et al. 2024; Yao et al. 2024). However, the co-role of oxidative stress and ferroptosis in SILI has not yet been studied, and further exploration is needed.

Wedelolactone (WEL) is a phytocoumarin isolated from traditional Chinese medicine. As a representative component, its hepatoprotective effect may be related to inhibiting oxidative stress, the inflammatory response, apoptosis and other pathways (Lu et al. 2016; Maya et al. 2018; Fan et al. 2021). However, the clinical application of WEL in treating SILI diseases is greatly limited by its insufficient targeting ability and poor solubility. Therefore, finding a potential drug delivery carrier to improve WEL's targeting is particularly important. The study found that macrophage-derived Exo can be targeted in animal liver (Fan et al. 2024), suggesting that macrophage derived Exo may be a promising targeted drug delivery vector for the treatment of SILI diseases. Therefore, the aim of this study was to construct a delivery system of Exos loaded with wedelolactone (Exo@WEL) to achieve efficient targeted delivery to the site of liver injury. On this basis, a systematic exploration will be carried out to evaluate the hepatoprotective effects of WEL and elucidate its underlying mechanisms of action. The findings are expected to provide novel therapeutic strategies and a theoretical foundation for treating SILI.

## 2. Materials and methods

### 2.1. Materials

1,1′-Dioctadecyl-3,3,3′,3′-Tetrame-thylindotricarbocyanine iodide (DiR) was supplied by Xi'an Ruixi Biological Technology Co. Ltd. (Xi'an, China). FITC anti-mice CD9 antibody and FITC anti-mice CD63 antibody were purchased from Beijing Biotechnology Co., Ltd. in China. CD81 monoclonal antibody (Eat2) and FITC purchased from Thermo Fisher in the USA. ROS purchased from Wuhan Saiwei Biotechnology Co., Ltd., ALT/AST, MDA/SOD, and GSH purchased from Nanjing Jiancheng Biotechnology Research Co., Ltd., TNF-α/IL-6/IL-1β were purchased from Quanzhou Ruixin Biotechnology Co., Ltd., HE staining solution was purchased from Wuhan Saiwei Co., Ltd., Nrf2, HO-1 purchased from Abcam.

### 2.2. Preparation of Exo

The macrophage (the mice macrophage cell line RAW264.7 provided by Haixing Biotechnology Co., Ltd.) derived Exo was defrosted at 37 °C and centrifuged at 4 °C 300 × *g* for 10 min to remove residual cells. The supernatant was subjected to gradient centrifugation successively: centrifugation at 4 °C and 3000 × *g* for 15 min to remove cell debris and centrifugation at 4 °C and 10,000 × *g* for 30 min to remove large particle vesicles. The supernatant was collected, transferred to a new tube, and centrifuged at 4 °C 110,000 × *g* for 70 min. After most of the supernatant was discarded, it was re-suspended with sterile PBS and filtered by 0.22 μm filter membrane. The filtrate (110,000 × *g*) was centrifuged for a second time for 70 min. Finally, the supernatant was abandoned and the precipitate at the bottom of the tube was suspended with pre-cooled PBS to obtain high purity Exo. After subpackaging, the filtrate was stored at −80 °C for a long time.

### 2.3. Characterization of Exo

10–20 μL Exo suspension drops were added to the electron microscope carrier net for 10 min. Excess droplets were absorbed and dried slightly, and 20 μL uranium acetate dye was added for 5 min. The dye was absorbed and dried by an incandescent lamp, and the images were observed under transmission electron microscope (TEM). The particle size and potential of Exo were detected by nanoparticle tracking analysis (NTA). In nanoflow detection, 30 μL Exo sample was mixed and diluted with 60 μL PBS. T 20 μL of fluorescently labeled antibody (CD9/CD63/CD81) was added, and the mixture was incubated at 37 °C for 30 min. Then, 1 mL pre-cooled PBS was added, and centrifuged at 110,000 × *g* at 4 °C for 70 min to remove the redundant antibody. After repeated washing, re-suspension with 50 μL PBS. After the instrument passes the standard performance test, pay attention to gradient dilution to avoid plugging the injection needle. After the sample is tested, the protein index result is obtained by NanoFCM(N30E) instrument.

### 2.4. Preparation and characterization of Exo@WEL

The Exo membrane structure is destroyed by ultrasonic mechanical vibration and cavitation effect to achieve efficient transmembrane drug delivery (Qi et al. 2023). The experiment was conducted according to the mass ratio of 8:1, 6:1, 5:1, 4:1, 2:1 and 1:1, and 6:1 was finally determined as the best ratio. The ultrasound was performed with 300 W power and 40 kHz frequency for 4 cycles (45 s on/2 min off, 5 min intercycle cooling). After ultrasonic treatment, the Exo film was incubated at 37 °C for 1 h in the dark to restore the stability of Exo film. Then transferred to 100 kDa ultrafiltration tube and centrifuged at 4000 rpm at 4 °C for 3 times (20 min each time) to remove the WEL (WEL is purchased from Baoji Chen Guang Biotechnology Co., Ltd.). Finally, Exo@WEL was obtained by aseptic PBS re-suspension precipitation. It was characterized by TEM, particle size, potential, and nanoflow detection (same method as characterization of Exo). *In vitro* release experiment, Exo@WEL was placed in a 3500 Da dialysis bag and incubated in a PBS sustained release system containing Tween 80 (100 rpm) at 37 °C. Regular sampling was performed, and an equal volume pre-warm fresh media was added. WEL release at different time points was analyzed by high-performance liquid chromatography (HPLC).

### 2.5. Establishment of HPLC analytical methodology

The content of WEL was determined by HPLC. The standard solution of 2.5–80 μg/mL (over-0.22 μm organic filter) was prepared by gradient dilution with 60% methanol + 40% 0.5% glacial acetic acid as the mobile phase (30 °C, 351 nm). A standard curve of peak area (Y) and concentration (X) was established (R² ≥ 0.999). The encapsulation rate (EE%) of Exo@WEL was quantitatively detected by HPLC, and the formula was calculated as EE% = (measured mass of WEL in Exo/total mass of initial administration) × 100%. The particle size and encapsulation rate at the 1st, 3rd, 5th, and 7th days were analyzed to evaluate the stability. The higher the EE value, the better the drug loading efficiency of Exo.

### 2.6. Induction and treatment of hepatic injury in sepsis

Male C57BL/6 mice (6–8 weeks old, 18–22 g) were obtained from the Air Force Medical University Laboratory Animal Center [Production License No. SYXK-(Shaan)-2019-001]. Mice were housed under specific pathogen-free (SPF) conditions with six animals per polycarbonate cage and maintained at 25 °C ± 2 °C with 50 ± 10% relative humidity under a 12-h light/dark cycle. All the animals had ad libitum access to standard laboratory chow and autoclaved water. Following one week of acclimatization, the mice were randomly allocated into four experimental groups (*n* = 20 per group) using a computer-generated randomization table. In this study, given that the CLP model caused the death of the mice, the initial number of mice in the CLP, CLP + WEL, and CLP + Exo@WEL groups was set at 20 to ensure that there were sufficient live animals available for sample collection and inter-group comparisons at the 24 h endpoint. All biochemical and molecular analyses were conducted only on the mice that survived until the endpoint. The sham group and CLP group were given food and water for daily use in the laboratory, while the drug administration group was given 600 μL WEL solution and Exo@WEL (WEL and Exo@WEL at the dose of 50 mg/kg for both) by intraperitoneal injection, respectively. First, a 1 h WEL/Exo@WEL pre-treatment is carried out. Then, the CLP surgery is performed. 24 h of observation were conducted after the surgery. That is, 1 h treatment → CLP → 24 h observation. The mice were euthanized 24 h post-surgery using the described protocol (the method we used was intraperitoneal injection, and the chemical substance used was sodium pentobarbital, with a dosage of 150 mg/kg). Liver tissues, serum, and plasma were then collected from all the experimental groups for subsequent analysis. The Fourth Military Medical University (Xi'an) Institutional Animal Care and Use Committee approved the animal care and procedures (IACUC-20230755).

### 2.7. Fluorescence imaging *in vivo* in mice

*In vivo*, fluorescence imaging was performed on C57BL/6 mice to evaluate the permeability of Exo@WEL to SILI. The mice were randomly divided into three groups: the Sham group, Exo group, and Exo@WEL group. Exo and Exo@WEL were labeled with the near-infrared fluorescent dye DiR (1 μM final concentration), and the dyes were mixed with Exo/Exo@WEL at a ratio of 1:100 (v/v). The dyes were incubated at 37 °C for 1 h without light. The labeling complex was obtained by removing unbound dyes with an Exo Spin Column (MWCO 100 kDa). The mice in the experimental group were injected intraperitoneally with fluorescently labeled Exo/Exo@WEL.Dynamic monitoring was performed by small animal imaging system at 0 h (baseline), 1 h, 2 h, 4 h, 8 h, 12 h, and 24 h after injection, respectively. The imaging parameters were set as follows: excitation wavelength, 748 nm; emission wavelength, 780 nm; exposure time, 500 ± 50 ms (automatic exposure mode), and spatial distribution and temporal dynamic changes in fluorescence signals were collected.

### 2.8. Imaging experiment of isolated major organs in mice

After live imaging was completed, the mice were killed under anesthesia. The major organs (heart, liver, spleen, lung, and kidney) were completely extracted and rinsed with normal saline. Then placed on a black imaging plate to eliminate background interference. A small animal imaging system (excitation/emission wavelength 748/780 nm) was used to collect fluorescence signals from isolated organs. The Living Image software was used to delimit regions of interest (ROI) for each organ, automatically deduct background fluorescence values of the same batch of unlabeled organs, and calculate standardized fluorescence intensity. In addition, quantitative analysis of *in vivo* fluorescence signals was performed by subtracting the background from the Sham group as the background control. Specifically, the average background intensity of the corresponding organs in each experimental group was subtracted from the ROI fluorescence intensity of that group. The data were reconstructed to generate the biological distribution heat map, which visually demonstrated the organ-targeting characteristics of Exo and Exo@WEL.

### 2.9. The distribution of Exo in liver was investigated by frozen fluorescence section

The distribution characteristics of Exo and Exo@WEL in liver tissues were analyzed by frozen section technique. After the *in vivo* imaging was complete, the mice were killed under anesthesia, and the livers were completely removed. The livers were rinsed with pre-cooling PBS for 3 times to remove residual blood. The surface moisture was absorbed by filter paper, and fixed in 4% paraformaldehyde fixing solution (4 °C away from light) for 24 h. After fixation, the liver tissue underwent gradient sucrose dehydration (15% and 30% for 24 h each), followed by OCT embedding and quick-freezing in liquid nitrogen. Frozen sections with a thickness of 10 μm were then prepared, mounted onto anti-slip slides, and stored at −80 °C. The distribution of the DiR fluorescence signal was observed by confocal microscopy (excitation wavelength, 748 nm; emission channel, 780 nm), with hepatocytes located via DAPI nuclear staining. Quantitative analysis was then performed to assess the differences in targeted accumulation between Exo and Exo@WEL in liver tissues.

### 2.10. Histopathologic section of liver

Liver tissue pathological sections were used to analyze the characteristics of the Sham group, CLP group, CLP + Exo group, and CLP + Exo@WEL group in liver tissues. Liver tissues from the mice were collected 24 h after CLP, rinsed with PBS and fixed in tissue fixative solution for 24 h. Dehydrated with gradient alcohol (low concentration to high concentration), treated with xylene transparent, and then made into 5 μm sections after paraffin embedding. The slices were flattened with hot water and attached to the slides, dried and dyed by HE: hematoxylin staining the nucleus for 5 min, acid water and ammonia water for 5 s, rinse with running water for 1 h, dehydrated with 70% and 90% alcohol for 10 min, eosin staining the cytoplasm for 3 min, and finally, anhydrous alcohol dehydration, xylene transparent, and neutral gum sealing. After natural drying, pathological damage in different areas of the liver tissue was observed under a microscope, and images were collected.

### 2.11. Exo@WEL safety investigation of animals after administration

After 24 h of drug administration, blood was taken from the eyeball and placed in an ordinary EP tube, left at room-temperature for 0.5–2 h for blood to coagulate naturally, centrifuged at 4 °C at 3000 rpm for 15 min, the upper serum was taken, and the biochemical function was detected by automatic biochemical analyzer. The main indicators were blood urea nitrogen (BUN) and creatinine (CREA). Blood was taken from the mouse orbit, placed in an anticoagulant tube, and centrifuged at 3000 rpm for 15 min, and the plasma part was taken for blood routine detection. The main detection indicators included white blood cell (WBC) and red blood cell (RBC). Pathological damage to the heart, liver, spleen, lung, and kidney was observed by HE staining using the 2.10 method to evaluate safety. Serum and plasma were collected from the control group and Exo@WEL group and analyzed with BUN, CREA, WBC, and RBC kits (Jiancheng, Nanjing, China).

### 2.12. Biochemical detection and microplate assay (ELISA)

The levels of AST (C010-2-1), ALT (C009-2-1), LDH (A020-2-2), SOD (A001-1), MDA (A003-1), and GSH (G435-48T) in the serum and the iron content (A039-2-1) in the liver tissue were detected by the kit. Serum IL-6 (GEM0001), TNF-α (GEM0004) and IL-1β (GEM0002) levels were measured by Servicebio ELISA kit. All the absorbance (OD) values were determined by Thermo Fisher Scientific spectrophotometer.

### 2.13. Immunofluorescence staining

The tissues or cells were divided into the Sham/Control group, CLP/LPS group, CLP/LPS + WEL group, and CLP/LPS + Exo@WEL group treated for immunofluorescence detection. After the liver tissue was fixed with 4% paraformaldehyde for 24 h, the 4μm sections were prepared by embedding dehydrated paraffin wax. After dewaxing, hydration, microwave antigen repair with citric acid, PBS washing 3 times (5 min each time), and 5%BSA sealing for 30 min. Nrf2, HO-1, SLC7A11 and GPX4 primary antibodies (1:200) were added, and incubated overnight at 4 °C. After washing with PBS, a fluorescent secondary antibody (1:200) of the corresponding species was added and incubated for 1 h in the dark, and DAPI was re-dyed for 10 min. The images were acquired by confocal microscopy through 405 nm (DAPI) and 488 nm (FITC) channels. Cell staining steps: The culture was inoculated in a confocal Petri dish at a density of 2 × 10^4^/mL, the LPS group was replaced with sugar-free serum-free DMEM medium, and the treatment group was incubated with WEL and Exo@WEL for 24 h, fixed with 4% paraformaldehyde for 20 min, permeated with 0.5% Triton X-100 for 20 min, and sealed with immunofluorescent blocking solution for 30 min. The Nrf2 primary antibody (1:200) was added overnight at 4 °C, and the fluorescent secondary antibody was incubated for 1 h in the dark. After nucleation by DAPI, fluorescence images were collected and analyzed by confocal microscopy.

### 2.14. Transmission electron microscopy (TEM)

After fixation with glutaraldehyde, the left liver leaves of the mice were dehydrated with gradient ethanol, and ultrathin sections were prepared and stained with lead citrate and uranium acetate. The cells were cultured overnight at a density of 10^6^/mL and then fixed with 4% paraformaldehyde for 2–4 h. After pre-embedding in agarose, the samples were post-fixed with 1% osmium tetroxide (in 0.1 M PBS, pH 7.4) under light-protected conditions, followed by room-temperature dehydration. Ultrathin sections were prepared after resin permeation and polymerization and then double-stained with uranyl acetate and lead citrate. Finally, a Hitachi transmission electron microscopy was used to observe and analyze.

### 2.15. Cell viability assay

The effects of WEL and Exo@WEL on the activity of AML12 (The normal mice liver cells line AML12 were purchased from Hai Xing Biotechnology Co., Ltd.) hepatocytes were evaluated by CCK-8 method. 1 × 10^4^ cells/mL single-cell suspension was inoculated in a 96-well plate (100 μL/well), and after 24 h of culture, WEL and Exo@WEL solution with gradient concentration (0.4–51.2 μg/mL) were added respectively (the effective dose of WEL in the Exo@WEL group was consistent with that in the WEL group), and 6 multiple wells were set in each group. After continuous culture for 24 h, the working liquid was prepared according to the medium: CCK-8 = 10:1 ratio, 110 μL was added to each hole (to avoid bubbles caused by the gun head contact with liquid surface), and PBS (100 μL/hole) was added to the outer ring of the hole plate to reduce edge effect. After incubation at 37 °C for 1–4 h for color development, the absorbance (OD) at 450 nm was determined with an enzyme-labeled instrument. Cell viability was calculated as follows: cell viability (%) = [(A experiment − A blank)/(A control − A blank)] × 100%, where experiment A was the OD value of the cell, drug and CCK-8 hole, blank A was the background value containing only medium + CCK-8, and control A was the positive control containing cell + CCK-8 (100% activity). The usage method of ML385 (S8790, an Nrf2 inhibitor) refers to similar published references (Liu et al. 2022). Specifically, the working concentration of ML385 was 10 μM. The drug was dissolved in dimethyl sulfoxide (DMSO) and diluted in complete medium to ensure that the DMSO concentration in the final culture system was less than 0.1% (v/v). After cell inoculation and culture for 24 h, the culture medium containing ML385 was added for further treatment for 24 h, and then the cells were collected to detect relevant indicators (such as signaling pathway proteins, reactive oxygen levels, or the mitochondrial membrane potential JC-1).

### 2.16. Cell uptake

The hydrophobic fluorescent dye DiR was used to label Exo and Exo@WEL, and DiR was mixed with 100 μg Exo at a volume ratio of 1:100. The free dyes were removed by Exo purification column after incubation at 37 °C for 1 h, and DiR labeled Exo and Exo@WEL were obtained. AML12 cells were seeded into confocal dishes and allowed to adhere overnight at 37 °C in a 5% CO2 incubator. DiR-labeled Exo@WEL was then added to the cells for co-culture under light-protected conditions, with time points set at 0, 6, 12, and 24 h (where the 0 h group served as an unincubated negative control). After the culture was terminated, the culture medium was absorbed and gently washed with pre-cooled PBS for 2–3 times to remove free Exo, and 4% paraformaldehyde was fixed at room-temperature for 30 min. After cleaning with PBS, the core was stained with DAPI (250 μL, 5 min away from light), and it was rinsed and sealed with PBS. Exo and Exo@WEL location were detected by 633 nm excitation light/660–690 nm emission light (DiR channel), and nuclei were displayed by 405 nm excitation light/430–460 nm emission light (DAPI channel). Cell uptake at different time points was quantitatively analyzed by multi-channel superposition imaging.

### 2.17. Mitochondrial membrane potential (MMP) test

The groups were divided into the control group, the LPS group, the LPS + WEL group and the LPS + Exo@WEL group for MMP analysis. After 24 h treatment, the cells were washed twice with PBS, and fresh medium containing JC-1 working solution (5 μL JC-1 probe + 1 mL staining buffer) was added. Incubate at 37 °C for 30 min without light. After the supernatant was abandoned, the JC-1 buffer was washed twice, and 2 mL of imaging medium was replaced. The emission signals at 590 nm (JC-1 polymer, red fluorescence, reflecting the normal mitochondrial membrane potential (MMP)) and 529 nm (JC-1 monomer, green fluorescence) were detected immediately by confocal microscopy under the excitation light of 488 nm. Complete the image acquisition within 30 min, and keep the incubator environment stable throughout the whole process.

### 2.18. Determination of ROS using a fluorescent probe

The DCFH-DA probe was diluted with serum-free medium at 1:1000 (1 mL working fluid per dish) according to the instructions. After discarding the original culture medium of each group of cells, the probe was gently washed twice with pre-warmed PBS to remove the residual medium. Add 1 mL of pre-prepared DCFH-DA working liquid (10 μM) to each dish, and incubate at 37 °C for 30 min. After incubation, the probe solution was discarded, and washed strictly with fresh serum-free medium three times (5 min each time) to completely remove the uninternalized free probe. Finally, the intracellular DCF fluorescence intensity was detected by laser confocal microscopy at 488 nm excitation wavelength, ROS specific green fluorescence signal was systematically collected, and multi-field images were taken for analysis. In order to ensure the stability of the probe, three multiple holes were set in each group, and the data were expressed by the relative fluorescence intensity (compared with the control group).

### 2.19. Western blot (WB) assay

Protein samples extracted from AML12 cells and liver tissues using RIPA lysis buffer (Beyotime Biotechnology, P002) containing protease and phosphatase inhibitors were separated by 10% SDS‒PAGE and transferred to PVDF membranes. After blocking with 5% skimmed milk for 1.5 h at room-temperature, the membranes were incubated overnight at 4 °C with primary antibodies against Nrf2 (1:800), GPX4 (1:800), SLC7A11 (1:800), HO-1 (1:800), or GAPDH (1:8000). Following incubation with HRP-conjugated secondary antibody (1:8000) for 1–2 h, the protein bands were detected using an ECL chemiluminescence kit (Abbkine) and visualized with a Nikon imaging system.

### 2.20. Statistical analysis

All the data were expressed as mean ± SD. GraphPad Prism 10.3.1 (GraphPad, San Diego, USA) was used to analyze the data. One-way ANOVA followed by Dunnett's post hoc test was performed to compare multiple groups. *p* < 0.05 or *p* < 0.01 was considered statistically significant.

## 3. Results

### 3.1. The preparation process of Exo@WEL

**Figure 1.**
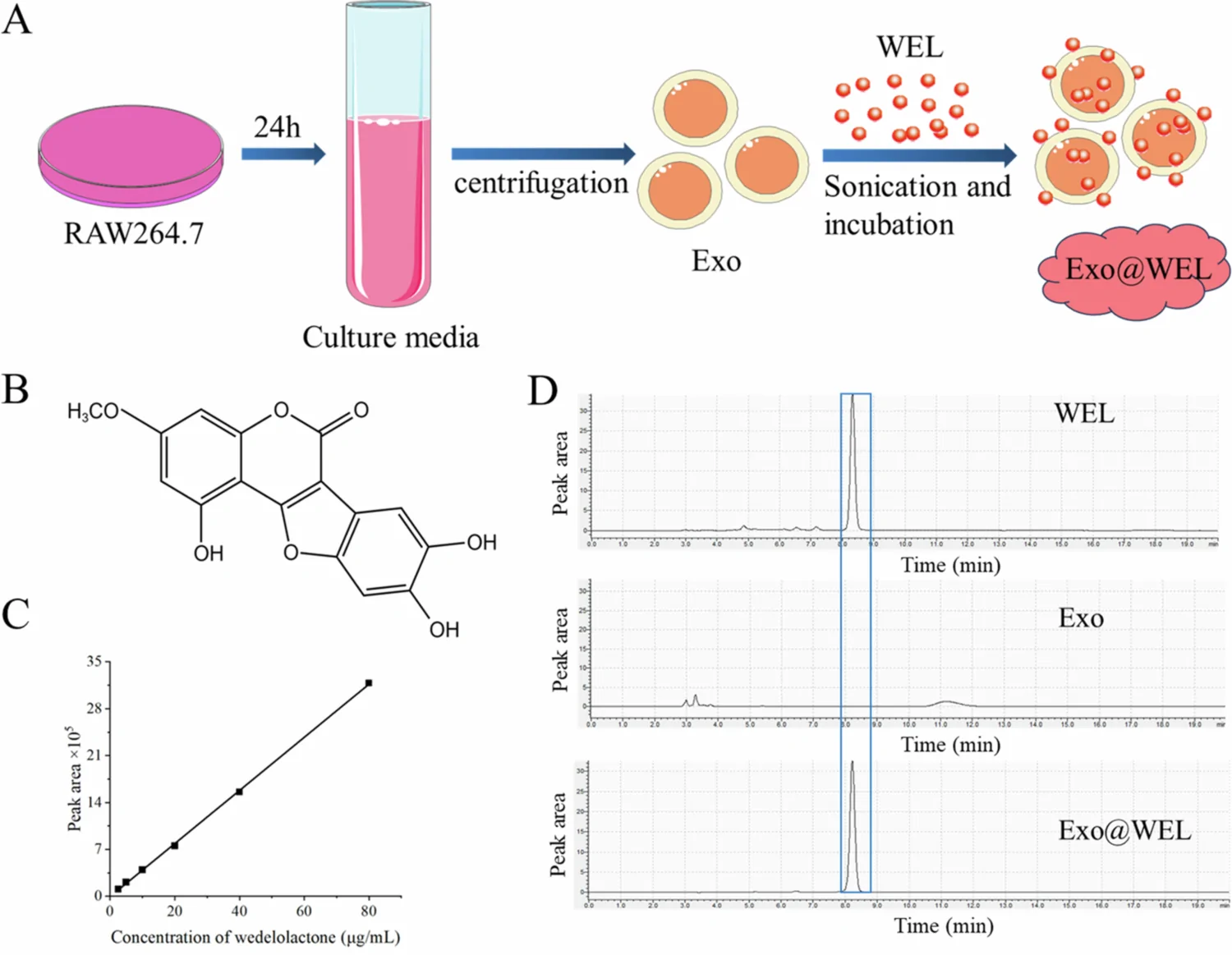
Characterization of Exo@WEL. (A) Preparation flow chart of Exo@WEL. (B) Chemical structure of WEL. (C) Standard curve of WEL (*n* = 3). (D) The HPLC results of Exo@WEL (*n* = 3).

The preparation process of Exo@WEL is shown in Figure 1A, which is composed of a mixture of macrophage-derived Exo and WEL according to a mass ratio of 6:1, and the chemical structure formula of WEL is shown in Figure 1B. In order to verify the synthesis effect, the WEL standard solution was first determined by HPLC, and a standard curve was drawn (Figure 1C). The results showed that the peak area (Y) of WEL presented a good linear relationship with the concentration (X) in the concentration range of 0~80 μg/mL, and the regression equation was Y = 39645X-6070 (R^2^ = 0.9996). Further HPLC analysis of WEL released by Exo@WEL showed that the peak time of WEL released from the broken film was completely consistent with that of the standard product (Figure 1D).

### 3.2. Preparation and characterization of Exo@WEL

**Figure 2.**
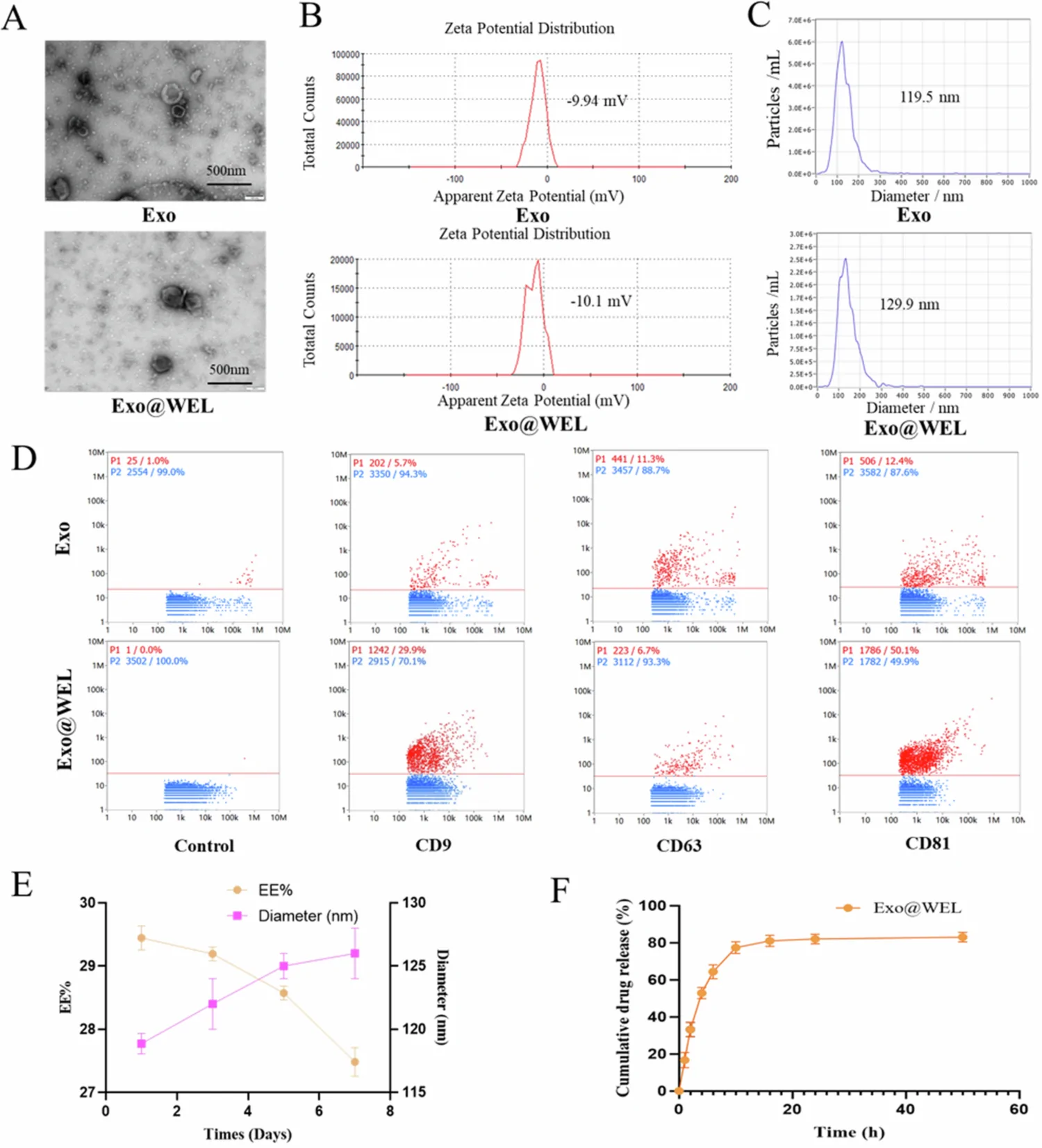
Exo characterization. (A) TEM images of Exo and Exo@WEL (scale bar = 500 nm). (B) Zeta potential distribution of Exo and Exo@WEL. (C) Particle size distribution of Exo and Exo@WEL. (D) Expression of the CD9, CD63, and CD81 proteins in Exo and Exo@WEL. (E) Stability of Exo@WEL (*n* = 3). (F) The cumulative WEL release from Exo@WEL in PBS solution (*n* = 3).

As illustrated in Figure 2A, TEM revealed the presence of characteristic vesicular structures in both Exo and Exo@WEL. The Zeta potential analysis, as depicted in Figure 2B, indicated that both samples exhibited a Zeta potential value of approximately −10 mV. Particle size distribution analysis demonstrated that both Exo and Exo@WEL maintained a particle size of around 120–130 nm, with a uniform distribution pattern (Figure 2C). Nanoflow cytometry detection, as shown in Figure 2D, confirmed the expression of the exosomal marker proteins CD9, CD63, and CD81 in both Exo and Exo@WEL. WEL characteristic peaks were detected by HPLC, confirming that the drug had been successfully loaded. Quantitative analysis further revealed an encapsulation efficiency and drug loading capacity of 25.97% ± 3.58% and 4.14% ± 0.14%, respectively. Stability testing over a period of seven days demonstrated that both the particle size and encapsulation efficiency of the formulation remained stable (Figure 2E). *In vitro* release experiments showed that the cumulative release of PBS reached 80% within 24 h (Figure 2F). These results indicate that the Exo@WEL prepared using this method possesses favorable physicochemical properties and stability, providing a robust foundation for its potential future applications.

### 3.3. *In vivo* biodistribution and liver targeting of Exo@WEL

**Figure 3.**
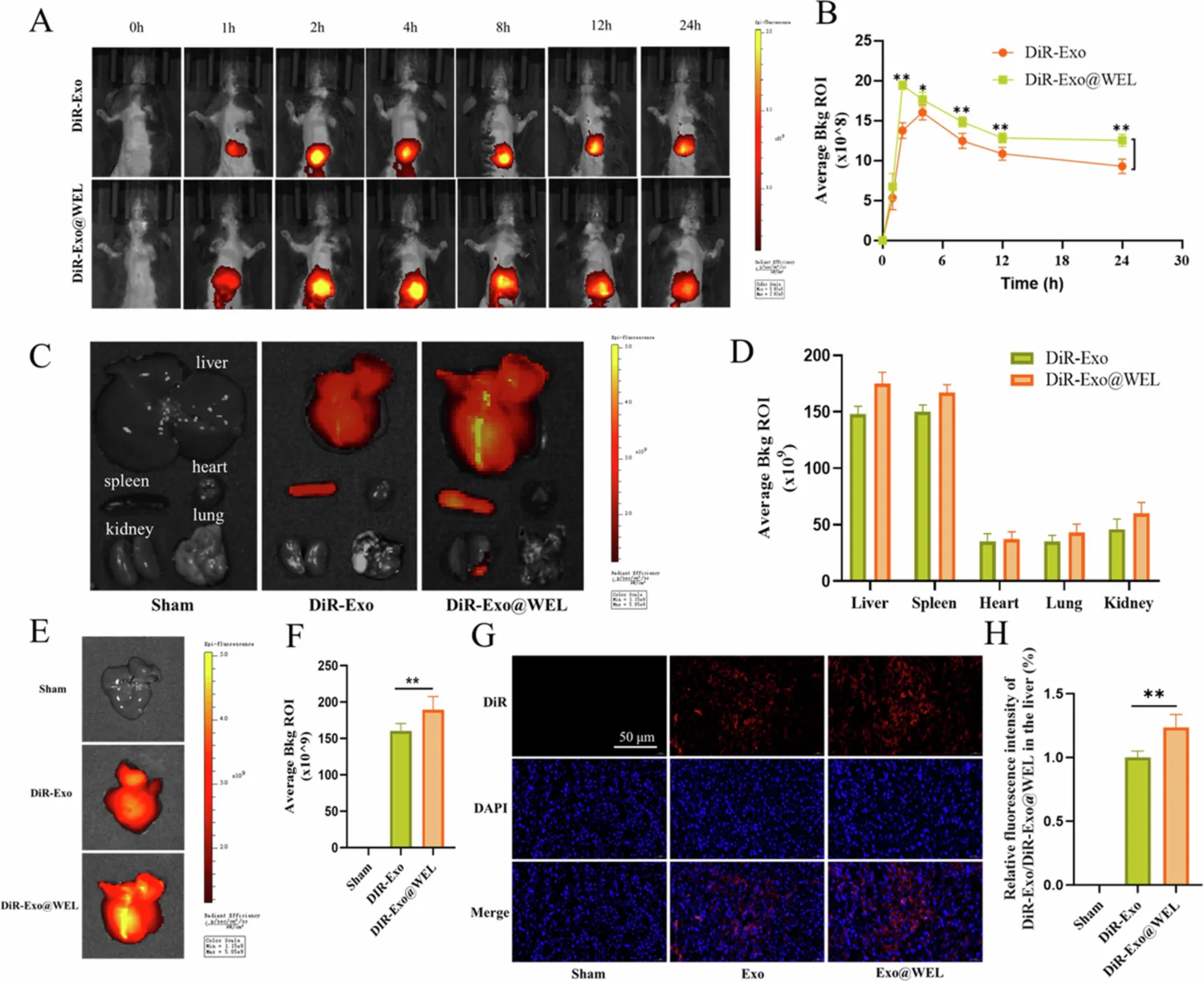
*In vivo* liver targeting of Exo. (A) Fluorescence analysis of Exo distribution in the liver tissue of the mice (B) Quantitative fluorescence results in mice liver within 24 h (*n* = 3, **p < 0.01, *p < 0.05). (C) Fluorescence analysis of Exo distribution in several main organs of mice, including the liver, spleen, heart, lung, and kidney. (D) Quantitative fluorescence results of the main organs (*n* = 3). (E) The mean fluorescence intensity in the liver of the mice. (F) Quantitative results of fluorescence in isolated liver (*n* = 3, **p < 0.01). (G) Targeting of Exo to liver of mice (scale bar = 50 μm). (H) Quantitative fluorescence results of Exo and Exo@WEL (*n* = 3).

In order to study the pharmacological efficacy and drug distribution characteristics of Exo *in vivo*. We labeled Exo and Exo@WEL with near-infrared fluorescent dye DiR for *in vivo* tracking purposes. As shown in Figure 3A and B, liver drug accumulation in the Exo@WEL group was significantly higher than that in the WEL group 24 h after administration (*p* < 0.01). Dynamic imaging shows that the enrichment of Exo@WEL in the liver accumulates rapidly, reaching the peak concentration at 2 h after injection, and then slowly decreases until significant signals can still be detected at 24 h. Further fluorescence detection and quantitative analysis were performed on the heart, liver, spleen, lung, kidney, and other major organs (Figure 3C and D), and it was found that Exo@WEL was the most prominent site of drug accumulation in the liver. Quantitative analysis of liver tissue showed that although drug residues existed in both treatment groups, drug accumulation was significantly higher in the Exo@WEL group (Figure 3E and F). To verify these findings, frozen sections of mice liver tissues taken 24 h after administration were analyzed, and the results showed that the intensity of the liver fluorescence signal in the Exo@WEL group was significantly higher than that in the Exo group (Figure 3G and H). These data consistently indicate that Exo@WEL can significantly enhance the targeted accumulation of drugs in the liver.

### 3.4. Safety validation and liver protection evaluation of Exo@WEL *in vivo*

**Figure 4.**
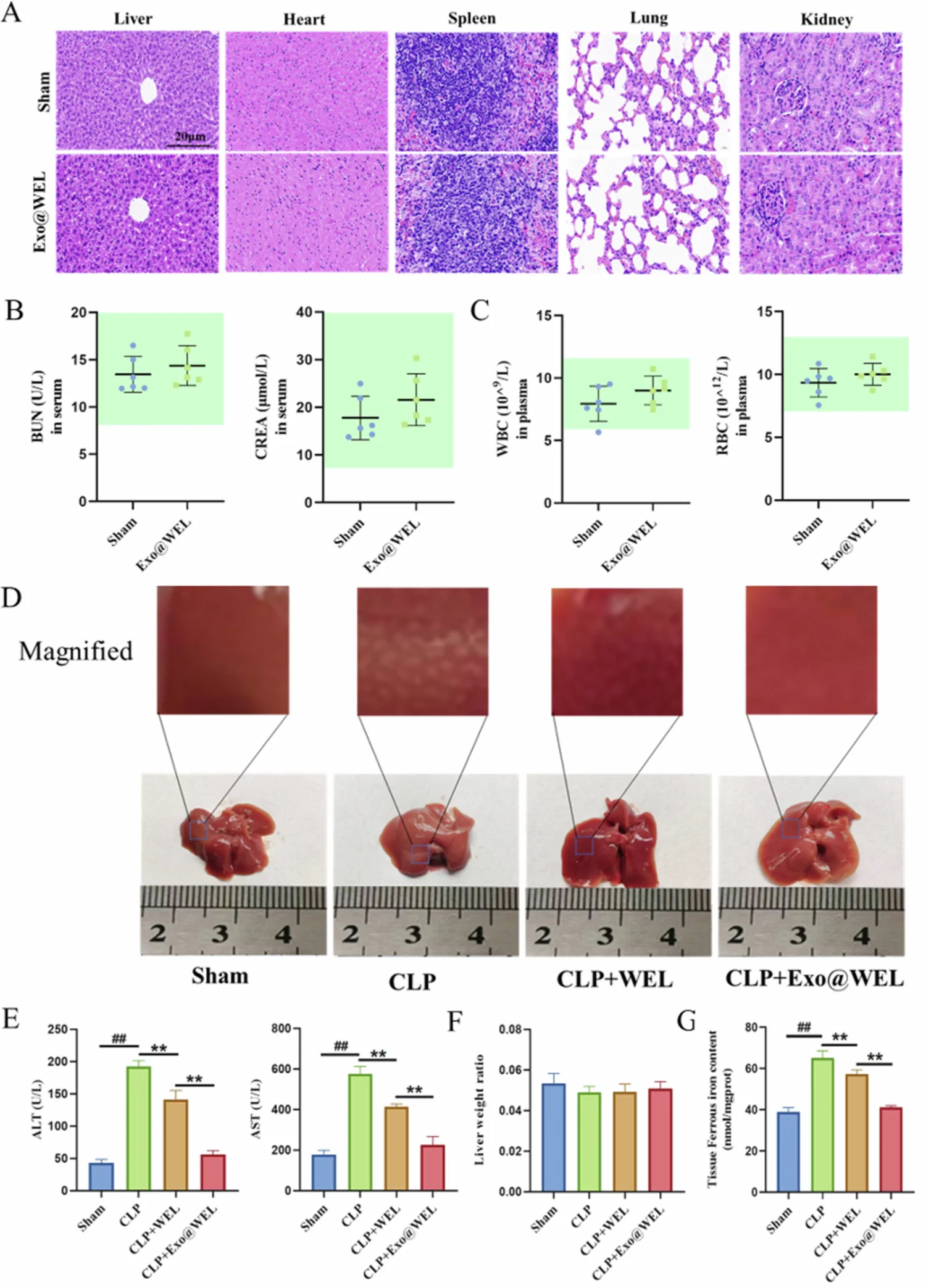
Safety assessment and therapeutic efficacy of Exo@WEL. (A) HE staining of liver, heart, spleen, lung, and kidney tissues of CLP mice (scale bar = 20 μm). (B and C) Effect of Exo@WEL on BUN and CREA activity in the serum of CLP mice; effect of Exo@WEL on BUN and CREA activity in the plasma of CLP mice. Green background indicates the normal range (*n* = 6). (D) Liver tissue morphology at 24 h post-CLP. (E) Effect of WEL and Exo@WEL on ALT and AST activity in the serum of CLP mice. (F) Liver weight ratio (24 h post-CLP) (*n* = 8). (G) Tissue ferrous iron content. (*n* = 6, ^##^ *p* < 0.01; *p < 0.05, **p < 0.01).

This study confirmed the safety of Exo@WEL intraperitoneal injection through multidimensional evaluation: histopathological analysis showed no structural damage in the liver, heart, spleen , lung, and kidneyof the mice after administration, and the cell arrangement was tight and complete (Figure 4A). As depicted in Figure 4B and C, the results of the blood biochemical examination indicated that renal function indicators, including blood urea nitrogen (BUN) and creatinine (CREA), as well as routine blood parameters (WBC, RBC), were all within the normal range. This finding confirmed that there were no adverse effects on the hematopoietic system. As shown in Figure 4D, the liver surface in the CLP group was rough and nodular, while the liver surface in the WEL and Exo@WEL groups was gradually smooth, with the most significant improvement in the Exo@WEL group. Figure 4E test shows that Exo@WEL can significantly reduce the elevation of CLP-induced aminotransferases (ALT and AST), and the effect is better than that of WEL group. As shown in Figure 4F, although the liver weight ratio decreased after CLP, there was no statistically significant difference among the treatment groups. As depicted in Figure 4G, Exo@WEL significantly alleviated CLP-induced intrahepatic iron accumulation.

### 3.5. Evaluation of antioxidant stress and inflammation efficacy of Exo@WEL *in vivo*

**Figure 5.**
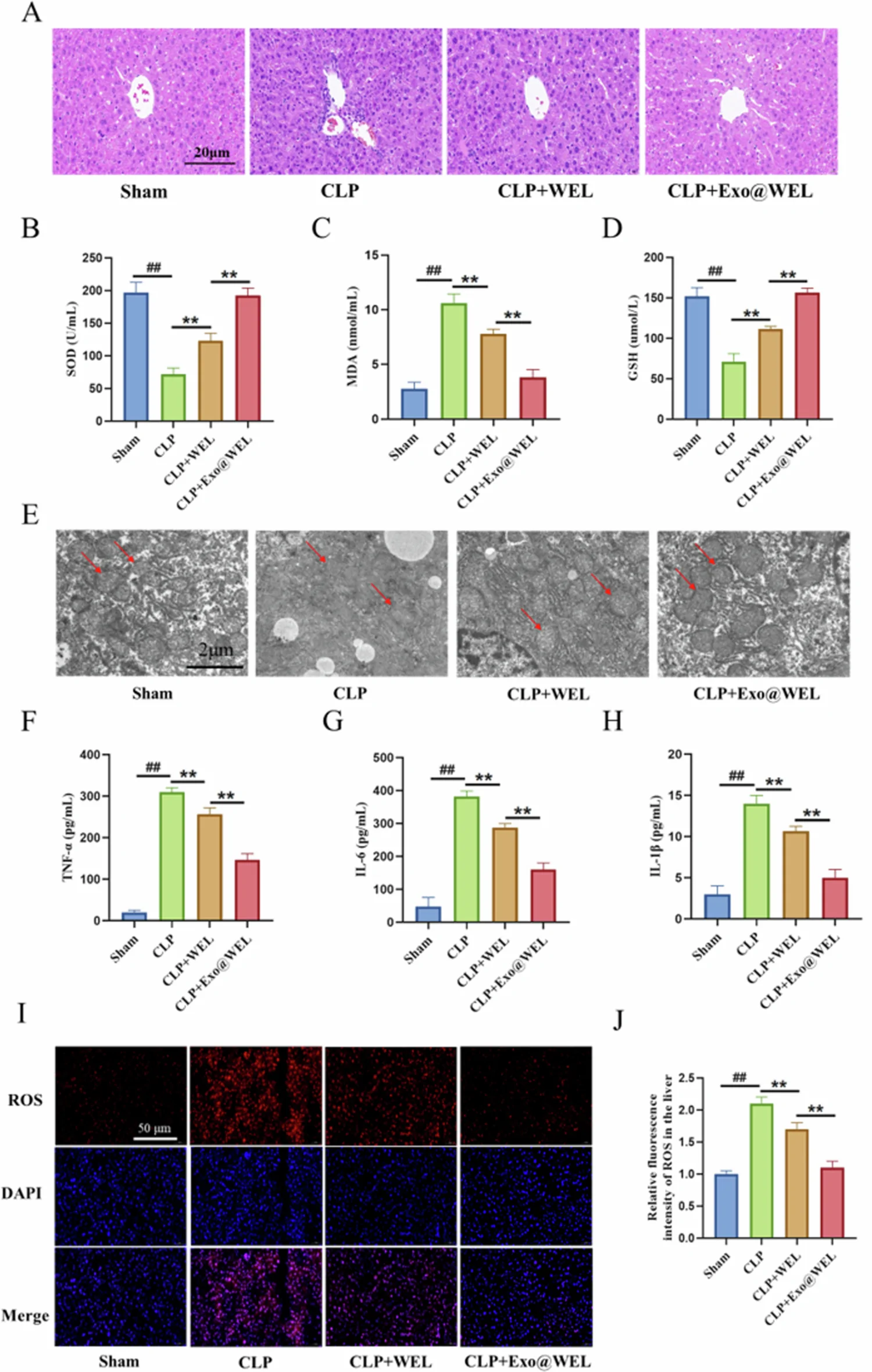
Exo@WEL can inhibit the inflammatory response and oxidative stress in mice. (A) The pathological changes of liver tissue under HE staining (scale bar = 20 μm). (B–D) The levels of SOD, MDA, and GSH in the serum (*n* = 6). (E) Changes in mitochondrial structure in liver tissue (scale bar = 2 μm). (F–H) The levels of TNF-α, IL-6, and IL-1β in the serum (*n* = 6). (I) Confocal detection of ROS fluorescence intensity in liver tissue (scale bar = 50 μm). (J) Relative ROS production. (*n* = 6, ^##^ *p* < 0.01; *p < 0.05, **p < 0.01).

To systematically validate the anti-inflammatory, antioxidant, and iron mortality effects of Exo@WEL, we conducted a multilevel experimental study. As shown in Figure 5A, compared with the Sham group with normal liver tissue structure, the CLP model group presented obvious hepatic cell arrangement disorder and inflammatory infiltration. WEL and Exo@WEL treatment groups could significantly improved these pathological changes. In terms of the oxidative stress indexes (Figure 5B–D), the CLP group showed typical ferroptosis characteristics: the levels of GSH and SOD were significantly reduced, and the content of MDA was significantly increased. WEL and Exo@WEL treatment can significantly reverse these changes, especially in the Exo@WEL group. As depicted in Figure 5E, TEM observation visualized that hepatocyte mitochondria in the CLP group showed obvious contraction, ridge breakage and other damage, and Exo@WEL treatment can effectively restore the normal morphology of the mitochondria. As shown in Figure 5F–H, the detection of inflammatory factors showed that Exo@WEL could significantly reduce the overrelease of TNF-α, IL-6, IL-1β and other pro-inflammatory factors induced by CLP. Immunofluorescence staining, as demonstrated in Figure 5I and J, indicated that Exo@WEL could significantly reduce the expression of ROS in liver tissue, thereby enhancing the antioxidant defense ability.

### 3.6. Exo@WEL alleviated liver injury via Nrf2/SLC7A11/GPX4 pathways in mice

**Figure 6.**
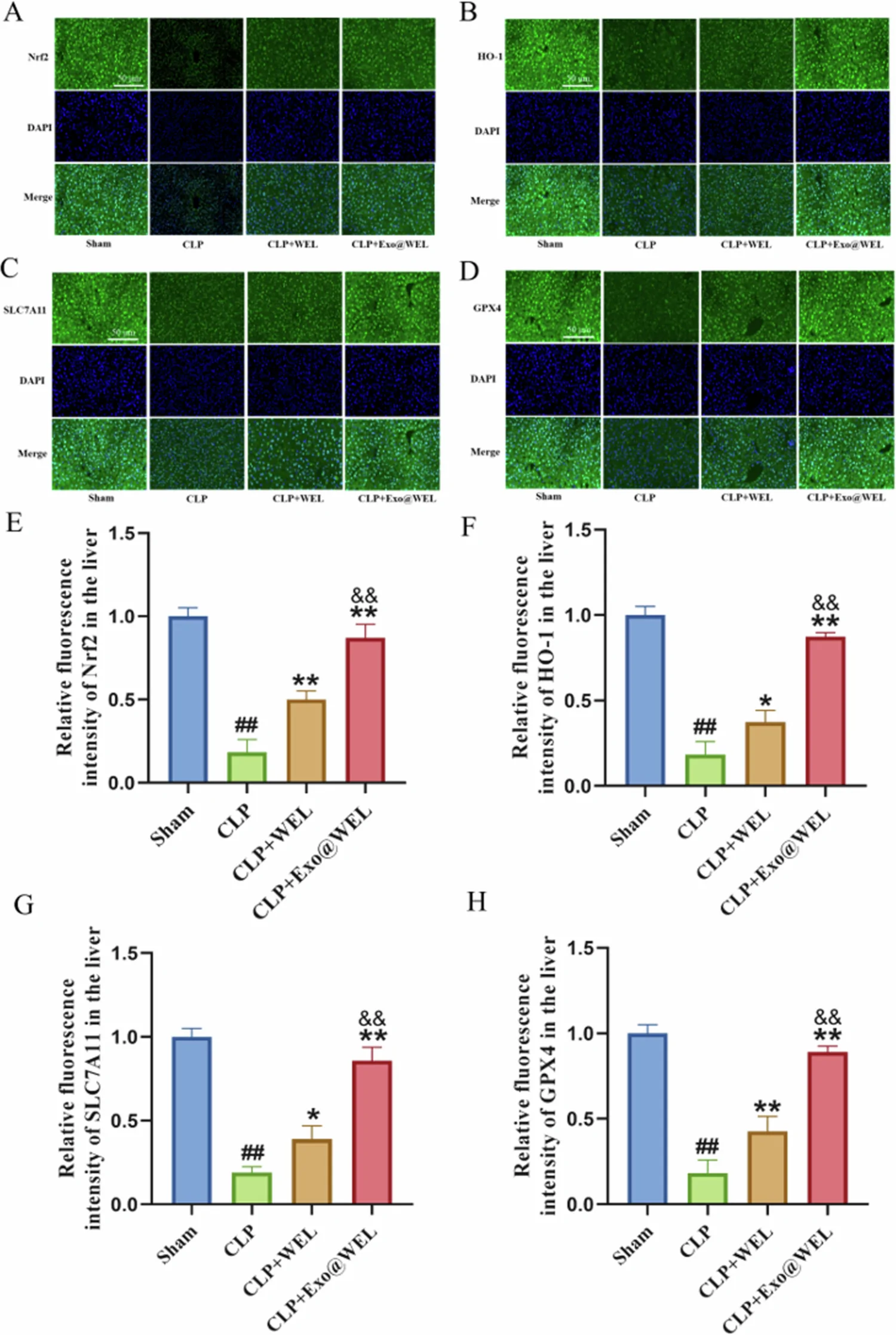
Exo@WEL can inhibit hepatic oxidative stress and ferroptosis in mice. (A–D) The expression levels of Nrf2, HO-1, SLC7A11 and GPX4 based on immunofluorescent staining (scale bar = 50 μm). (E–H) Statistical analysis in the expression levels of Nrf2, HO-1, SLC7A11 and GPX4 based on immunofluorescent staining (*n* = 3). (I) The expression levels of Nrf2, HO-1, SLC7A11 and GPX4 were detected by Western blot. (J–M) Statistical analysis in the expression levels of Nrf2, HO-1, SLC7A11 and GPX4. (*n* = 3, ^##^ *p* < 0.01; *p < 0.05, **p < 0.01).

To elucidate the molecular mechanisms underlying the Exo@WEL antioxidant and anti-ferroptosis effects *in vivo*, we performed comprehensive protein expression analyses in CLP-induced liver injury models. Immunofluorescence and Western blot results consistently demonstrated that Exo@WEL treatment significantly modulated key ferroptosis-related signaling pathways (Figure 6A–M). Compared to WEL treatment, Exo@WEL significantly upregulated the expression of key ferroptosis-inhibiting proteins (Nrf2, HO-1, SLC7A11, and GPX4). This regulation of the Nrf2/SLC7A11/GPX4 pathway effectively suppressed ferroptosis and oxidative damage in hepatic tissues. The enhanced bioactivity of Exo@WEL underscores the therapeutic potential of Exo-based delivery systems for multi-target intervention in liver injury.

### 3.7. Cellular uptake

**Figure 7.**
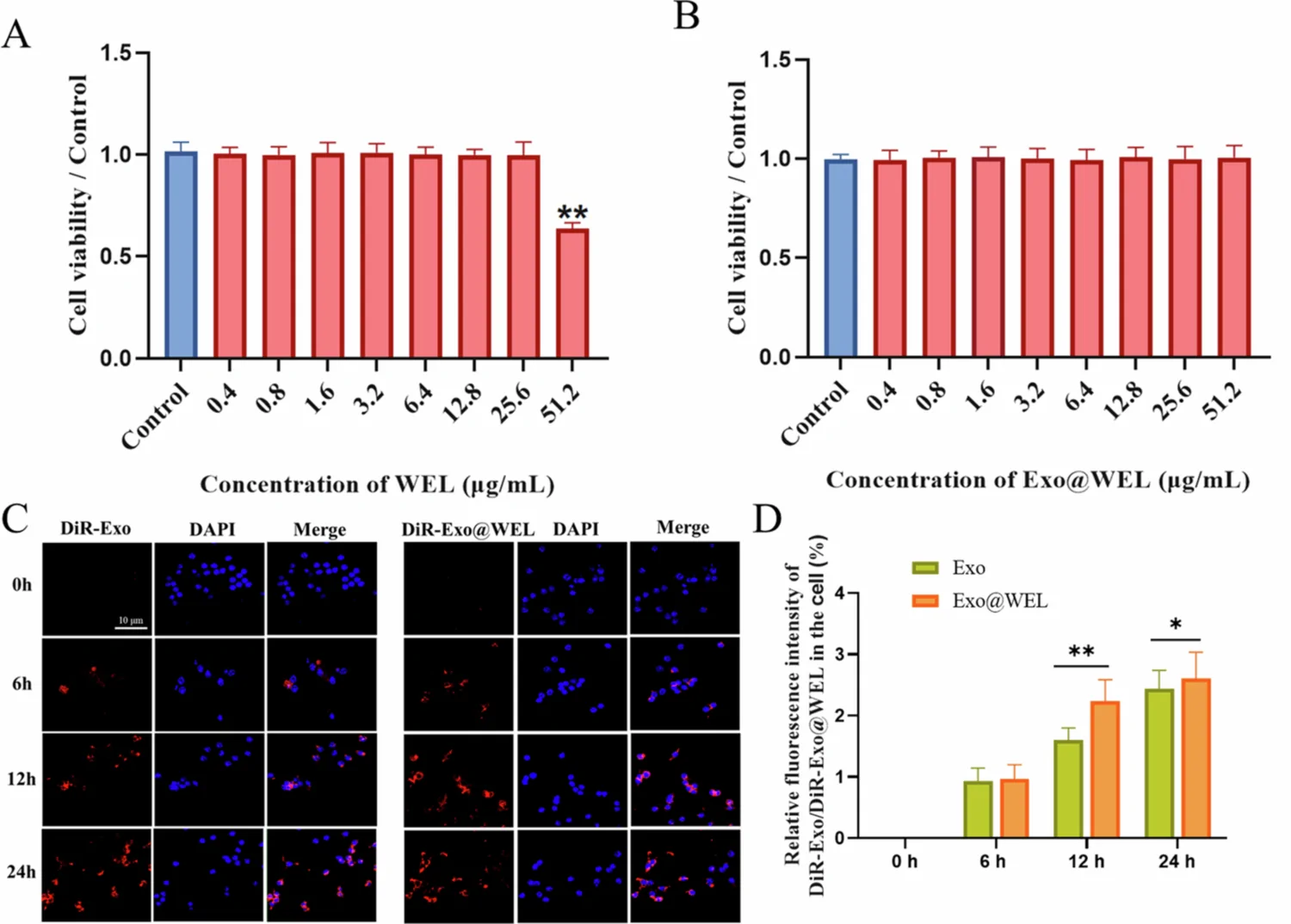
Cell uptake of Exo. (A and B) Toxic effects of WEL and Exo@WEL at different concentrations on AML12 cells (*n* = 6). (C) The uptake of DiR-labelled Exo in LPS-treated AML12 cells was determined by CLSM (scale bar = 10 μm). (D) Quantitative analysis of the fluorescence value of cell uptake. (*n* = 3, *p < 0.05, **p < 0.01).

The cellular uptake characteristics and biocompatibility of Exo@WEL were evaluated. Cytotoxicity showed that WEL and Exo@WEL had no significant impact on cell viability in 0–25.6 μg/mL. However, when the concentration rose to 51.2 μg/mL, WEL showed significant cytotoxicity, while Exo@WEL maintained good biocompatibility (Figure 7A and B). Based on this, 25.6 μg/mL was selected as the working concentration of Exo@WEL for subsequent experiments. Confocal laser microscopy showed that DiR-labeled Exos and Exo@WEL showed time-dependent uptake in AML12 cells (6 h, 12 h, 24 h), and the fluorescence intensity of the Exo@WEL group was significantly higher than that of the Exo group (Figure 7C and D). The results showed that Exo@WEL not only significantly enhanced the enrichment efficiency of Exo in hepatocytes but also provided an important basis for their application in the treatment of liver diseases.

### 3.8. Exo@WEL can reduce sepsis-induced liver injury and oxidative stress in cell

**Figure 8.**
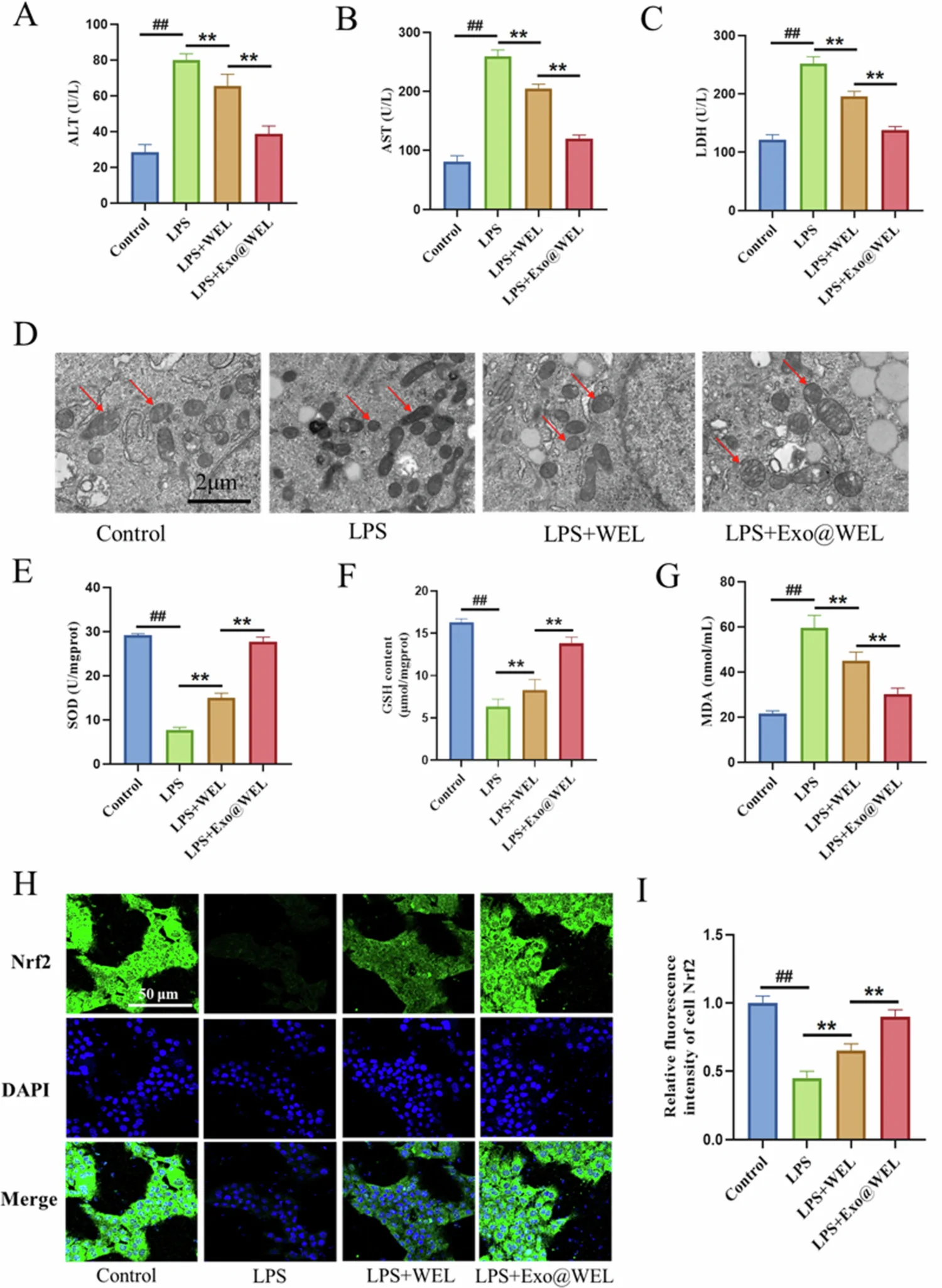
Exo@WEL can alleviate liver cell injury in sepsis. (A–C) Cell supernatant levels of ALT, AST and LDH (*n* = 6). (D) Mitochondrial changes observed by TEM (scale bar = 2;μm) (*n* = 3). (E–G) Cell supernatant levels of SOD, MDA and GSH (*n* = 6). (H) The expression level of Nrf2 was detected by immunofluorescence staining (scale bar = 50 μm). (I) Statistical analysis in the expression levels (*n* = 3, ^##^ *p* < 0.01; **p < 0.01).

The results showed that Exo@WEL has an effective therapeutic effect on LPS-induced liver injury. Compared with the control group, LPS significantly increased the levels of ALT, AST and LDH in AML12 cells, and WEL and Exo@WEL significantly decreased the levels of ALT, AST and LDH in AML12 cells, with the better effect on Exo@WEL (Figure 8A–C). As depicted in Figure 8D, ultrastructural analysis Exo@WEL effectively alleviated LPS-induced mitochondrial damage, including ridge destruction and membrane coagulation. As illustrated in Figure 8E–G, biochemical experiments confirmed that Exo@WEL has antioxidant ability by restoring SOD/GSH and reducing MDA. Immunofluorescence staining demonstrated that Exo@WEL upregulated Nrf2 expression in hepatocytes (Figure 8H and I).

### 3.9. Exo@WEL alleviated liver injury via Nrf2/SLC7A11/GPX4 pathways in cell

**Figure 9.**
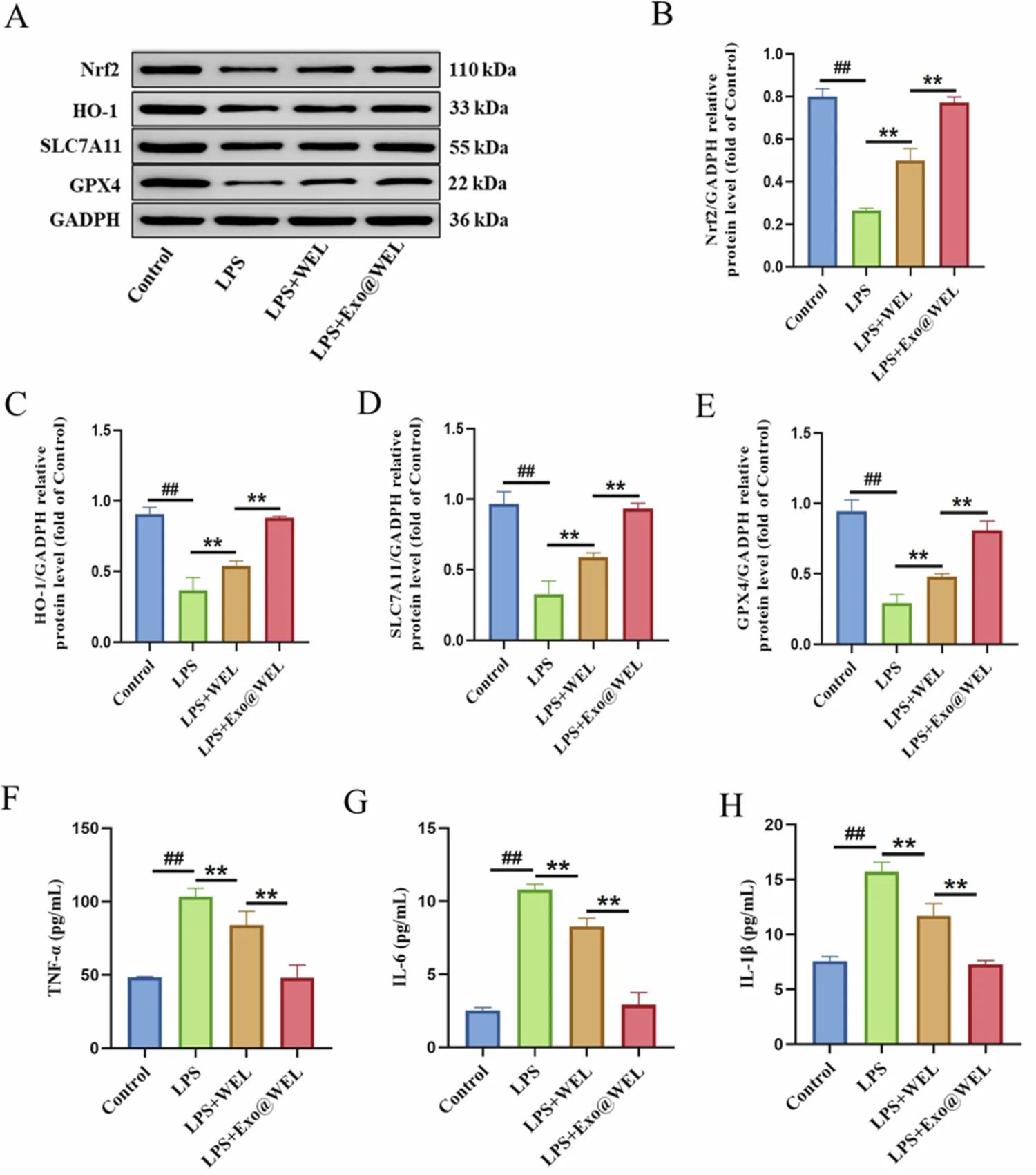
Exo@WEL can inhibit hepatic oxidative stress and ferroptosis in AML12 cells. (A) The expression levels of Nrf2, HO-1, SLC7A11,and GPX4 were detected by Western blot. (B–G) Statistical analysis in the expression levels of statistica (*n* = 3). (H–J) Cell supernatant levels of TNF-α, IL-6, and IL-1β (*n* = 6, ^##^ *p* < 0.01; **p < 0.01).

As illustrated in Figure 9A–E, analysis revealed that LPS stimulation caused severe pathological lesions. WEL and Exo@WEL both effectively reverse these pathological changes by upregulating antioxidant protein, with Exo@WEL showing significantly better outcomes for all parameters. According to Figure 9F–H, the levels of inflammatory cytokines further confirmed the therapeutic effect. Compared with WEL, Exo@WEL showed a significantly enhanced inhibitory effect on the LPS-induced elevation of TNF-α, IL-6, and IL-1β levels. These consistent molecular and cellular findings suggest that Exo@WEL coordinates the Nrf2/SLC7A11/GPX4 signaling pathways while reducing oxidative stress, ferroptosis, and inflammation, thereby providing overall protection against SILI.

### 3.10. Inhibition of Nrf2 partially suppressed Exo@WEL activity in suppressing hepatocyte ferroptosis

**Figure 10.**
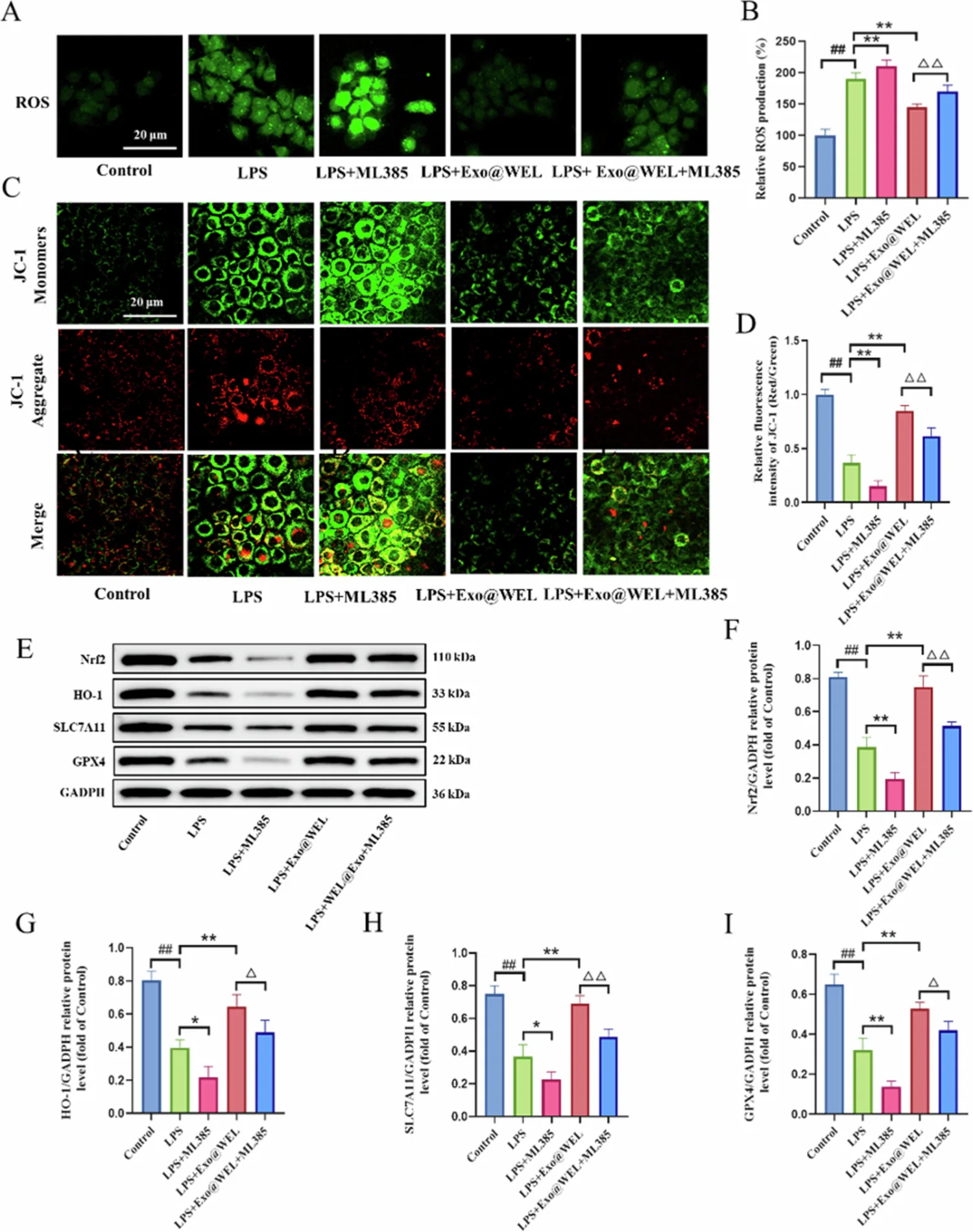
Inhibition of Nrf2 partly abolished Exo@WEL activity in suppressing hepatocyte ferroptosis. (A) Confocal detection of ROS fluorescence intensity (scale bar = 20 μm). (B) Relative ROS production. (C and D) Detection of MMP damage in AML12 cells using a JC-1 probe (scale bar = 20 μm). (E–I) The expression of GPX4, SLC7A11, HO-1, and Nrf2 detected by western blotting and fold activation data analysis (*n* = 6, ^##^ *p* < 0.01; *p < 0.05, **p < 0.01; *^∆^p* < 0.05, ^*∆∆*^^*p*^< 0.01).

To elucidate the pivotal protective mechanism of Nrf2 in SILI and the mechanism of action of Exo@WEL, we conducted verification experiments by adding the Nrf2 inhibitor ML385. The findings demonstrated that LPS treatment significantly increased ROS levels in hepatocytes. Moreover, the addition of ML385 further exacerbated oxidative stress. In contrast, Exo@WEL was capable of significantly suppressing the expression of ROS. However, ML385 partially nullified this inhibitory effect of Exo@WEL (Figure 10A and B), suggesting that Exo@WEL exerts its antioxidant role by activating Nrf2. JC-1 fluorescence probe detection showed that ML385 not only aggravated LPS-induced MMP damage but also weakened the protective effect of Exo@WEL on the MMP (Figure 10C and D). Western blot analysis (Figure 10E–I) further showed that ML385 significantly down-regulated the expression of Nrf2 and its downstream antioxidant proteins (HO-1, SLC7A11, GPX4), while Exo@WEL could reverse the LPS-induced down-regulation of these proteins. The results showed that Exo@WEL regulates the antioxidant defense system and key protein of iron deposition by activating the Nrf2 signaling pathway, thereby alleviating oxidative stress and iron deposition reaction, and providing a new molecular mechanism basis for SILI therapy.

## 4. Discussion

WEL, a plant-based coumarin isolated from *Eclipta prostrata* (a traditional Chinese medicine), exhibits significant liver-protective effects (Yin et al. 2025), but its clinical application is severely limited due to poor solubility and insufficient targeting. Aiming at the current clinical challenges of SILI therapy, such as the short duration of action and lack of liver-specific targeting (Lu et al. 2023), we constructed an Exo@WEL nanodelivery system. This system gives full play to the advantages of Exo with good biocompatibility, low immunogenicity and natural targeting, and effectively overcomes the limitations of WEL. Experiments have confirmed that Exo@WEL has a significant enrichment ability in the liver and that its therapeutic effect is significantly better than that of WEL, providing an important basis for the development of targeted nanodelivery systems based on Exo.

Exo, as the frontier carrier platform in the field of nanomedical medicine, have become an important tool for targeted therapy for cancer, cardiovascular diseases and infectious diseases because of its natural targeting, biosafety and multi-functional loading capacity (Fang et al. 2024; Zhang et al. 2024; Wang et al. 2025). In particular, Exo derived from macrophages not only have tissue repair function, but also have the characteristics of high biocompatibility and low immunogenicity (Cui et al. 2023; Pan et al. 2023; Zhang et al. 2024), making them an ideal choice for small-molecule drug carriers. In this study, Exo derived from unstimulated M0 macrophages were used. The nanoscale vesicles secreted in the resting state carry abundant bioactive molecules, which can truly reflect the basic biological characteristics of macrophages and avoid experimental bias caused by changes in the polarization state, making them ideal carriers for WEL loading (Zhang et al. 2025). Exo and Exo@WEL were characterized by TEM, NTA, and nanoflow. TEM showed that both of them had typical vesicular structures. NTA analysis shows that the particle size of Exo is concentrated at about 120 nm and increases slightly to about 130 nm after WEL loading, which is consistent with literature reports (Kanchanapally et al. 2019). Nanoflow detection confirmed that both of them expressed the Exo marker proteins CD9, CD63 and CD81, which were in line with the guidelines of the International Extracellular Vesicle Association (Małys et al. 2021). Exo were derived from the mice macrophage cell line RAW264.7 and isolated via ultracentrifugation. Subsequently, the Exo@WEL was subsequently prepared from these Exo using ultrasonic incubation. The prepared Exo@WEL achieved an encapsulation rate of 20%-30%. Furthermore, the encapsulation rate obtained from this preliminary study still has significant space for improvement. Future work will focus on process optimization to enhance the encapsulation rate and drug loading rate, laying the foundation for potential translational applications. Significantly, all the characterization data verified that the loading of WEL did not substantially change the inherent properties of the Exo. This indicates that Exo serve as an optimal carrier for WEL, and Exo-based drug delivery systems exhibit promising potential for SILI treatment. However, the detailed molecular mechanisms of Exo@WEL still require further investigation.

In addition, the distribution characteristics of Exo@WEL *in vivo* were systematically evaluated by DiR near infrared fluorescence labeling technique. The research results show that the drug reaches its peak concentration within 2 h, and compared with Exo, Exo@WEL exhibits a more significant targeted enrichment effect in the liver, and the drug remains in the body for a longer period of time. The rapid liver-targeting and effective pre-treatment achieved at the onset of sepsis are among the reasons why we designed this delivery system. The enhanced liver targeting efficacy of Exo@WEL is likely attributed to the successful loading of WEL, which could alter the Exo's physicochemical profile and consequently its interaction with the biological environment (Walker et al. 2019; Herrmann et al. 2021). Future studies employing techniques such as zeta potential measurement and surface proteomic analysis are warranted to directly characterize these modifications and elucidate the precise mechanism. At the cellular level, it was found that Exo@WEL had good biocompatibility with AML12 hepatocytes. Specifically, CCK-8 experiment showed that WEL inhibited cell activity at a concentration of 51.2 μg/mL, whereas Exo@WEL at the same dose had minimal impact on cell activity. Moreover, confocal laser microscopy analysis showed that cell uptake of Exo/Exo@WEL increased in a time-dependent manner.

The *in vivo* experiment selects the CLP gold standard model to evaluate the overall efficacy (Dejager et al. 2011; Wu et al. 2025). the *in vitro* experiment selects the classic LPS stimulation model to analyze the cellular mechanism (Wang et al. 2024; Long et al. 2025). This design aimed to jointly verify the protective effects of Exo@WEL from different levels. In both the *in vivo* and *in vitro* SILI models, Exo@WEL demonstrated significant therapeutic effects. HE staining and liver function indices (AST and ALT) revealed its effectiveness in alleviating hepatocyte injury and inflammatory infiltration. The treatment simultaneously reduced the levels of oxidative stress markers (MDA and ROS) while increasing the expression of antioxidant enzymes (SOD and GSH). Additionally, it improved the mitochondrial structure and significantly inhibited the production of pro-inflammatory factors (IL-1β, IL-6, and TNF-α). Compared to WEL group, the Exo@WEL group exhibited better therapeutic efficacy because of the targeting advantages of the Exo delivery system. Comprehensive safety assessments confirmed Exo@WEL's excellent systemic safety and biocompatibility. Furthermore, this study mainly provided preliminary imaging evidence of the liver-targeting property of Exo@WEL. This discovery laid the foundation for further in-depth pharmacokinetic studies. To comprehensively and quantitatively evaluate its performance as a drug delivery system, it is necessary to obtain key parameters such as its blood half-life, clearance rate, total systemic exposure (AUC), and Cmax. Constructing complete key parameters of pharmacokinetics provides indispensable data support for clinical translation. This study adopted the preventive strategy of pre-administration 24 h before modeling to verify the targeting accumulation ability and prevention potential of Exo@WEL. Although this provided clear evidence for mechanism research, its clinical translation requires further evaluation of its efficacy within the therapeutic administration window after the occurrence of sepsis. Future research will focus on the therapeutic effects of Exo@WEL given at different time points after CLP surgery (such as 1, 3, and 6 h) to determine its clinically applicable treatment time window.

From the mechanism level analysis, this study uncovered that the activation of Nrf2 and induce the transcription of downstream ferroptosis-related target genes (such as GPX4, SLC7A11 and HO-1), which play a dual role in anti-oxidative stress and anti-ferroptosis in sepsis (Zhang et al. 2023). In addition, Nrf2 also reduces intracellular iron accumulation and lipid peroxidation by regulating proteins related to iron metabolism, thereby inhibiting ferroptosis (Dodson et al. 2019). Specifically, Nrf2, on the one hand, promotes the metabolic transformation of free iron, on the other hand, it clears the lipid peroxidation products caused by iron overload, and maintains iron homeostasis. Studies have confirmed that Nrf2 becomes a negative regulator of ferroptosis by up-regulating the expression of key proteins such as GPX4 and SLC7A11 (Van Der Poll et al. 2017). In this study, the expression of Nrf2 was significantly down-regulated in the SILI group, and Exo@WEL significantly enhances SLC7A11-mediated cystinine uptake (for GSH synthesis) and GPX4-dependent lipid peroxide clearance through activation of the Nrf2 pathway, thereby blocking the ferroptosis process (Kanchanapally et al., 2019; Zhang et al. 2025). This mechanism suggests that targeted regulation of the Nrf2 pathway and its downstream effector molecules (such as GPX4 and SLC7A11) may provide a new strategy for the treatment of iron-related diseases and that Exo@WEL shows significant anti-ferroptosis and liver protection potential through multi-target synergism.

To delve deeper into the role of Nrf2 in SILI and the anti-ferroptosis mechanism of Exo@WEL, a series of experiments were conducted using the Nrf2 inhibitor ML385. The results showed that after ML385 treatment of AML12 cells with iron-mediated cell death, the intracellular ROS content was significantly increased, mitochondrial function was severely impaired, and the protein expression levels of Nrf2, HO-1, SLC7A11, and GPX4 were significantly upregulated. These results strongly imply that Nrf2 plays a crucial regulatory role in the context of SILI. Furthermore, ML385 significantly was found to significantly reduce the expression levels of Nrf2, SLC7A11, HO-1, and GPX4, and Exo@WEL partially up-regulated the expression levels of Nrf2, SLC7A11, HO-1, and GPX4. This additional evidence further validates the conclusion that Exo@WEL alleviates ferroptosis by activating the SLC7A11/GPX4 signaling pathway. Furthermore, the results of this study indicate that Nrf2 inhibitors can partially reverse this protective effect, suggesting that the Nrf2 pathway is crucial but not the only mechanism. In fact, other signaling pathways (such as NF-κB, apoptosis, autophagy, etc.) also play a key role in organ damage caused by sepsis. Studies have shown that Clemastine can alleviate sepsis-induced kidney injury through the α-Klotho/TLR-4/MYD-88/NF-κB/Caspase-3/p-P38 MAPK pathway and reduce liver injury through the GSDMD/NLRP-3/Caspase-1/NF-κB pathway (Abdelnaser et al. 2025; 2025). While Gabapentin can also alleviate SILI by regulating NF-κB/MAPK signaling, and improve kidney injury through the Nrf2/HO-1 and apoptosis pathways (Abdelnaser et al. 2023; 2024). The results of this study indicate that the Exo delivery system (Exo@WEL) demonstrates a significant protective effect on sepsis-induced multi-organ damage, which is consistent with the recent trend of the successful application of various antioxidant and anti-inflammatory combined strategies in sepsis models. For instance, the combined use of melatonin and ascorbic acid has been proven to effectively alleviate heart, kidney, and lung injuries caused by sepsis (Üstündağ et al. 2023; 2023; Abdelnaser et al. 2024), suggesting that the combined intervention of antioxidant and anti-inflammatory mechanisms holds significant value in the protection of multiple organs in sepsis. Similarly, the combined application of coenzyme Q10 and vitamin C has also shown a synergistic protective effect in the heart injury model induced by CLP (Üstündağ et al. 2024). These studies collectively indicate that combined therapy based on multiple targets and mechanisms has broad prospects in the treatment of sepsis. Compared with the aforementioned small-molecule combined therapies, Exo@WEL not only integrates the antioxidant and anti-inflammatory activities of WEL but also enhances the drug's enrichment in damaged organs through the natural targeting characteristics of Exo, thereby achieving a more persistent protective effect at a lower dose. Future research can further explore the combination strategies of Exo@WEL with other organ-protective agents (such as melatonin, coenzyme Q10, etc.) to optimize comprehensive treatment plans for multi-organ dysfunction in sepsis.

Collectively, we demonstrate that Exo@WEL effectively inhibits iron-mediated ferroptosis and oxidative stress by regulating dual signaling pathways and at the same time reduce the release of inflammatory factors, thus protecting hepatocytes from sepsis-induced damage. These findings provide new ideas and potential therapeutic targets for SILI.

## 5. Conclusions

**Figure 11.**
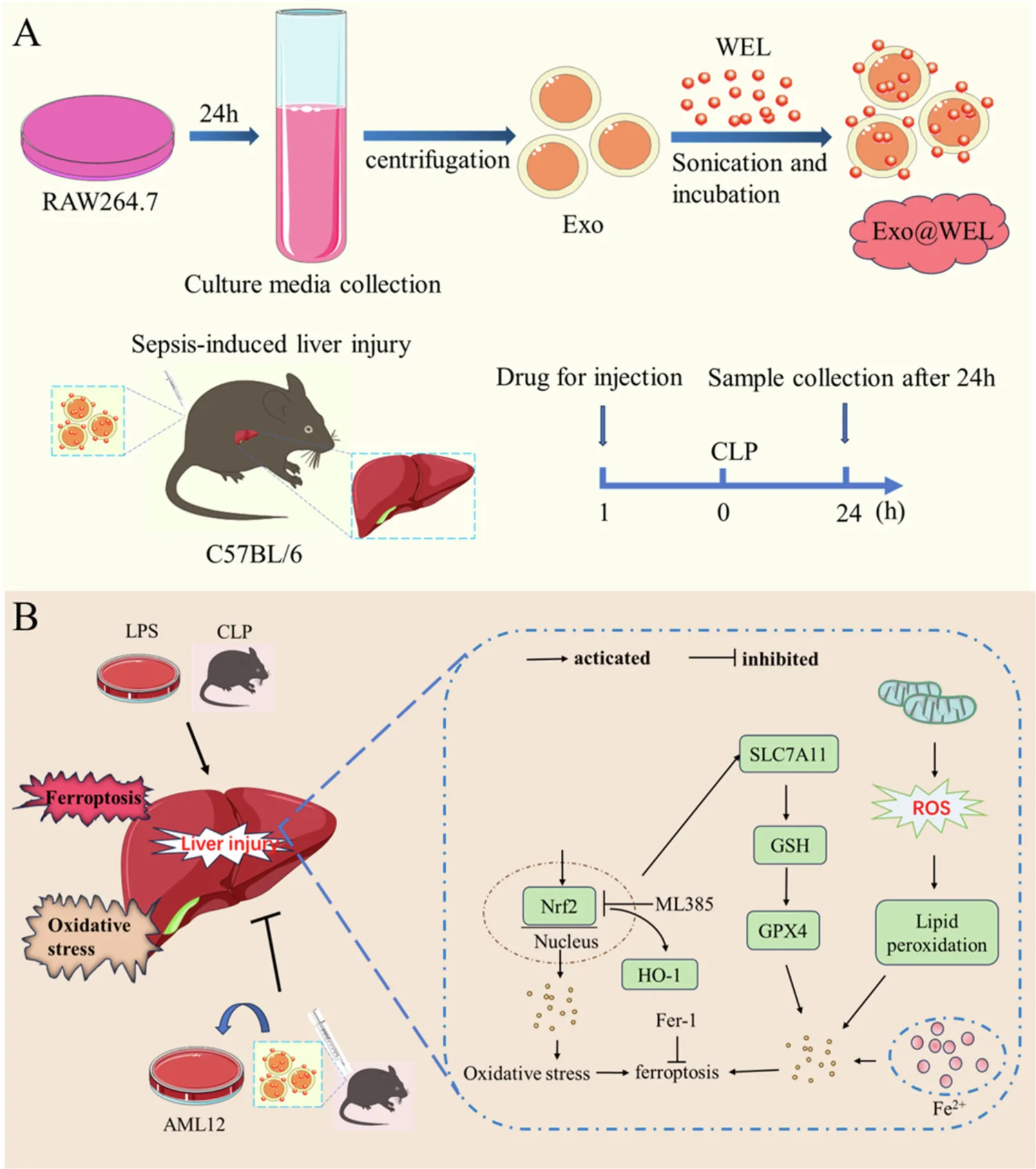
The scheme and potential mechanism of Exo@WEL were constructed. (A). Schematic diagram of Exo@WEL synthesis. (B). Mechanisms of action of Exo@WEL on SILI and hepatocytes in mice.

In summary, this study successfully developed a liver-targeted Exo delivery system (Exo@WEL), which significantly improved the therapeutic effect against SILI. Exo@WEL demonstrated a time-dependent increase in uptake efficiency by AML12 hepatocytes *in vitro*, while *in vivo* studies revealed liver-specific accumulation in mice, prolonging drug retention and elevating hepatic drug concentrations. This study is a pre-administration study, and this design is for the purpose of verifying the mechanism. Pharmacodynamic evaluation demonstrated that this system significantly enhanced hepatocyte survival rates and inhibit oxidative stress and ferroptosis through coordinated regulation of the Nrf2/SLC7A11/GPX4 signaling pathways (Figure 11). These findings not only establish a novel therapeutic strategy for SILI but also provide a critical framework for advancing Exo-based targeted drug delivery systems.

## Supplementary Material

Supplementary Material Author_Checklist_Full_Wedelactone_Loaded_Exosomes_for_Sepsis_Induced_Liver_Injury_A_Novel.pdf

## Data Availability

The data that support the findings of this study are available from the corresponding author, Mufei, upon reasonable request.
