# Correction

**DOI:** 10.1080/10717544.2026.2722441

**Published:** 2026-08-20

**Article Title:** Wedelactone-Loaded Exosomes for Sepsis-Induced Liver Injury: A Novel Therapeutic Strategy.

**Authors:** Yin, Y., Zhang, L., Yin, Y., Zhao, J., Gong, R., Zhou, X., … Wang, J.

**Journal:** *Drug Delivery*

**Bibliometrics:** Volume 33, Number 01, pages 1-26

**DOI:** http://doi.org/10.1080/10717544.2026.2693353

The article was originally published with incorrect Figure 6.

Upon receiving the revised Figure, the article was republished with updated Figure 6.

