# Supplementary material for: Wedelactone-loaded exosomes for sepsis-induced liver injury: a novel therapeutic strategy
Source: Drug Deliv. 2026 Jun 30;33(1):2693353. doi: 10.1080/10717544.2026.2693353 (PMC13495035; doi:10.1080/10717544.2026.2693353)
Supplement: Supplementary file 1 [file IDRD_A_2693353_SM2510.pdf]

NOTE: Please save this file locally before filling in the table, DO NOT work on the file within your internet browser as changes will not be saved. Adobe Acrobat Reader (available free here) is recommended for completion.

The ARRIVE guidelines 2.0: author checklist

The ARRIVE Essential 10

These items are the basic minimum to include in a manuscript. Without this information, readers and reviewers cannot assess the reliability of the findings.

Item		Recommendation	Section/line number, or reason for not reporting
**Study design**	1	For each experiment, provide brief details of study design including:	
		a. The groups being compared, including control groups. If no control group has been used, the rationale should be stated.	Immunofluorescence staining/ line 28-29 Page 9 of 115
		b. The experimental unit (e.g. a single animal, litter, or cage of animals).	Induction and treatment of hepatic injury in sepsis/ line 43 Page 6 of 115
**Sample size**	2	a. Specify the exact number of experimental units allocated to each group, and the total number in each experiment. Also indicate the total number of animals used.	Induction and treatment of hepatic injury in sepsis/ line 47 Page 6 of 115
		b. Explain how the sample size was decided. Provide details of any *a priori* sample size calculation, if done.	Induction and treatment of hepatic injury in sepsis/ line 48-52 Page 6 of 115
**Inclusion and exclusion criteria**	3	a. Describe any criteria used for including and excluding animals (or experimental units) during the experiment, and data points during the analysis. Specify if these criteria were established *a priori*. If no criteria were set, state this explicitly.	All procedures in this study were carried out in accordance with the protocol approved by the Animal Ethics Committee of Air Force Military Medical University (Approval Number: SYXK(Shaanxi)2019-001), and followed the ethical guidelines for animal research
		b. For each experimental group, report any animals, experimental units or data points not included in the analysis and explain why. If there were no exclusions, state so.	During the entire experiment, a total of 3 animals were excluded from the final analysis for the following reasons: CLP group: 11 mice died within 1 h after CLP surgery, and massive bleeding was found in the abdominal
		c. For each analysis, report the exact value of *n* in each experimental group.	At the beginning of the experiment, 20 animals were randomly assigned to each group (n = 20). The number of qualified animals in each group that entered the data statistical analysis; Sham (n = 20), CLP (n =
**Randomisation**	4	a. State whether randomisation was used to allocate experimental units to control and treatment groups. If done, provide the method used to generate the randomisation sequence.	Induction and treatment of hepatic injury in sepsis/ line 47 Page 6 of 115
		b. Describe the strategy used to minimise potential confounders such as the order of treatments and measurements, or animal/cage location. If confounders were not controlled, state this explicitly.	Induction and treatment of hepatic injury in sepsis/ 41-56 Page 6 of 115 and 4-7 Page 7 of 115
**Blinding**	5	Describe who was aware of the group allocation at the different stages of the experiment (during the allocation, the conduct of the experiment, the outcome assessment, and the data analysis).	To minimize the subjective bias in the experimental operation and result evaluation process to the greatest extent, this study implemented blinding at key stages of the entire experimental process. The awareness of the grouping information at each stage is as follows: Allocation stage (randomization grouping): Yanping Yin, who was responsible
**Outcome measures**	6	a. Clearly define all outcome measures assessed (e.g. cell death, molecular markers, or behavioural changes).	Preparation and characterization of Exo@WEL/ Figure 4 D-G
		b. For hypothesis-testing studies, specify the primary outcome measure, i.e. the outcome measure that was used to determine the sample size.	Induction and treatment of hepatic injury in sepsis/ line 48-55
**Statistical methods**	7	a. Provide details of the statistical methods used for each analysis, including software used.	Statistical analysis/ line 21-25
		b. Describe any methods used to assess whether the data met the assumptions of the statistical approach, and what was done if the assumptions were not met.	Statistical analysis/ line 21-25
**Experimental animals**	8	a. Provide species-appropriate details of the animals used, including species, strain and substrain, sex, age or developmental stage, and, if relevant, weight.	Induction and treatment of hepatic injury in sepsis/ line 38-45
		b. Provide further relevant information on the provenance of animals, health/immune status, genetic modification status, genotype, and any previous procedures.	Induction and treatment of hepatic injury in sepsis/ line 38-45
**Experimental procedures**	9	For each experimental group, including controls, describe the procedures in enough detail to allow others to replicate them, including:	
		a. What was done, how it was done and what was used.	
		b. When and how often.	Please refer to "a" for details.
		c. Where (including detail of any acclimatisation periods).	Air Force Military Medical University
		d. Why (provide rationale for procedures).	It can be verified for the efficacy of the medicine.
**Results**	10	For each experiment conducted, including independent replications, report:	
		a. Summary/descriptive statistics for each experimental group, with a measure of variability where applicable (e.g. mean and SD, or median and range).	The preparation process of Exo@WEL\\
		b. If applicable, the effect size with a confidence interval.	(n = 6, *P < 0.05, **P < 0.01).

The Recommended Set

These items complement the Essential 10 and add important context to the study. Reporting the items in both sets represents best practice.

Item		Recommendation	Section/line number, or reason for not reporting
**Abstract**	11	Provide an accurate summary of the research objectives, animal species, strain and sex, key methods, principal findings, and study conclusions.	line 10-39 Page 2 of 115
**Background**	12	a. Include sufficient scientific background to understand the rationale and context for the study, and explain the experimental approach.<br>b. Explain how the animal species and model used address the scientific objectives and, where appropriate, the relevance to human biology.	Introduction/ Page 2 of 115<br>Discussion/ line 26-28 Page 29 of 115
**Objectives**	13	Clearly describe the research question, research objectives and, where appropriate, specific hypotheses being tested.	Abstract/ line 10-20 Page 2 of 115
**Ethical statement**	14	Provide the name of the ethical review committee or equivalent that has approved the use of animals in this study, and any relevant licence or protocol numbers (if applicable). If ethical approval was not sought or granted, provide a justification.	Ethical approval statement/ Page 32 of 115
**Housing and husbandry**	15	Provide details of housing and husbandry conditions, including any environmental enrichment.	Induction and treatment of hepatic injury in sep
**Animal care and monitoring**	16	a. Describe any interventions or steps taken in the experimental protocols to reduce pain, suffering and distress.<br>b. Report any expected or unexpected adverse events.<br>c. Describe the humane endpoints established for the study, the signs that were monitored and the frequency of monitoring. If the study did not have humane endpoints, state this.	a. To maximize animal welfare, alleviate their suffering, and ensure the reliability of scientific data, the following intervention measures and monitoring plans were formulated and strictly implemented in this study. Analgesia and anesthesia: Before the CLP, all mice in the<br>b. Expected adverse events: The CLP surgery itself can cause sepsis, and it is expected tha the animals will exhibit reduced activity, piloerection, curling, etc., and even severe bleeding or death due to infection. Animal loss: Due to the severity of the CLP model, to<br>c. Humanely induced endpoint criteria: If any of the following indicators occur in the animals before the predetermined 24 h endpoint, euthanasia will be immediately carried out: significant weight loss (more than 20% of the initial weight); severe respiratory difficulties;
**Interpretation/ scientific implications**	17	a. Interpret the results, taking into account the study objectives and hypotheses, current theory and other relevant studies in the literature.<br>b. Comment on the study limitations including potential sources of bias, limitations of the animal model, and imprecision associated with the results.	Discussion/ line<br>Discussion/ line 45-60
**Generalisability/ translation**	18	Comment on whether, and how, the findings of this study are likely to generalise to other species or experimental conditions, including any relevance to human biology (where appropriate).	The results of this study are not only effective under specific experimental conditions, but also their core findings and strategy design provide important bases and clear directions for application in other species, models, and eventual clinical translation. 1) This study reveals that inhibiting ferroptosis and oxidative stress through activating the Nrf2/SLC7A11/GPX4 signaling axis has high
**Protocol registration**	19	Provide a statement indicating whether a protocol (including the research question, key design features, and analysis plan) was prepared before the study, and if and where this protocol was registered.	Before conducting the animal experiments, this study had already formulated a detailed experimental plan. This plan clearly defined the research question, the core hypothesis, the key design features (including the animal model, grouping, intervention measures) and the main analysis plan. However, this experimental plan was not prospectively registered on the publicly available and
**Data access**	20	Provide a statement describing if and where study data are available.	All the original data generated by this research
**Declaration of interests**	21	a. Declare any potential conflicts of interest, including financial and non-financial. If none exist, this should be stated.<br>b. List all funding sources (including grant identifier) and the role of the funder(s) in the design, analysis and reporting of the study.	line 53-60 Page 33 of 115<br>line 42-50 Page 33 of 115
